# Knockdown of AKT3 Activates HER2 and DDR Kinases in Bone-Seeking Breast Cancer Cells, Promotes Metastasis In Vivo and Attenuates the TGFβ/CTGF Axis

**DOI:** 10.3390/cells10020430

**Published:** 2021-02-18

**Authors:** Nico Hinz, Anke Baranowsky, Michael Horn, Malte Kriegs, Freya Sibbertsen, Daniel J. Smit, Philippe Clezardin, Tobias Lange, Thorsten Schinke, Manfred Jücker

**Affiliations:** 1 Center for Experimental Medicine, Institute of Biochemistry and Signal Transduction, University Medical Center Hamburg-Eppendorf, 20246 Hamburg, Germany; hinz_nico@gmx.de (N.H.); f.sibbertsen@uke.de (F.S.); d.smit@uke.de (D.J.S.); 2 Center for Experimental Medicine, Department of Osteology and Biomechanics, University Medical Center Hamburg-Eppendorf, 20246 Hamburg, Germany; a.baranowsky@uke.de (A.B.); schinke@uke.de (T.S.); 3 Department of Trauma and Orthopedic Surgery, University Medical Center Hamburg-Eppendorf, 20246 Hamburg, Germany; 4 University Cancer Center Hamburg, University Medical Center Hamburg-Eppendorf, 20246 Hamburg, Germany; mi.horn@uke.de; 5 Mildred Scheel Cancer Career Center Hamburg, University Medical Center Hamburg-Eppendorf, 20246 Hamburg, Germany; 6 Department of Radiotherapy & Radiation Oncology, University Medical Center Hamburg-Eppendorf, 20246 Hamburg, Germany; m.kriegs@uke.de; 7 UCCH Kinomics Core Facility, University Cancer Center Hamburg, University Medical Center Hamburg-Eppendorf, 20246 Hamburg, Germany; 8 INSERM, Research Unit UMR S1033, LyOS, Faculty of Medicine Lyon-Est, University of Lyon 1, 69372 Lyon, France; p.clezardin@sheffield.ac.uk; 9 Center for Experimental Medicine, Department of Anatomy and Experimental Morphology, University Medical Center Hamburg-Eppendorf, 20246 Hamburg, Germany; to.lange@uke.de

**Keywords:** AKT, AKT isoforms, breast cancer, metastasis, bone metastasis, vicious cycle, organ tropism, osteolysis

## Abstract

Bone metastases frequently occur in breast cancer patients and lack appropriate treatment options. Hence, understanding the molecular mechanisms involved in the multistep process of breast cancer bone metastasis and tumor-induced osteolysis is of paramount interest. The serine/threonine kinase AKT plays a crucial role in breast cancer bone metastasis but the effect of individual AKT isoforms remains unclear. Therefore, AKT isoform-specific knockdowns were generated on the bone-seeking MDA-MB-231 BO subline and the effect on proliferation, migration, invasion, and chemotaxis was analyzed by live-cell imaging. Kinome profiling and Western blot analysis of the TGFβ/CTGF axis were conducted and metastasis was evaluated by intracardiac inoculation of tumor cells into NOD scid gamma (NSG) mice. MDA-MB-231 BO cells exhibited an elevated AKT3 kinase activity in vitro and responded to combined treatment with AKT- and mTOR-inhibitors. Knockdown of AKT3 significantly increased migration, invasion, and chemotaxis in vitro and metastasis to bone but did not significantly enhance osteolysis. Furthermore, knockdown of AKT3 increased the activity and phosphorylation of pro-metastatic HER2 and DDR1/2 but lowered protein levels of CTGF after TGFβ-stimulation, an axis involved in tumor-induced osteolysis. We demonstrated that AKT3 plays a crucial role in bone-seeking breast cancer cells by promoting metastatic potential without facilitating tumor-induced osteolysis.

## 1. Introduction

Breast cancer is the most frequent cancer entity among women. Moreover, it causes the second highest rate of cancer-related death [1]. This tumor entity preferably metastasizes to bone, lung, liver, and brain, of which bone is the most common distant site [2]. Bone metastases cause bone pain, hypercalcemia, and pathological fractures and therefore, compromise quality of life in patients [3]. Once bone metastases emerge, breast cancer disease is incurable and a supportive treatment with anti-resorptive bisphosphonates or monoclonal antibodies such as denosumab is the only remaining option [4]. Breast cancer is divided into subtypes by identification of positivity for estrogen receptor, progesterone receptor, and HER2 (ErbB2 receptor tyrosine kinase 2). The triple negative breast cancer (TNBC) subtype is defined as hormone receptor and HER negative and exhibits a particularly high invasiveness [5]. Although bone metastases are more likely to occur in hormone receptor or HER2 positive breast cancer subtypes, patients with bone metastases of TNBC show the shortest median survival among all breast cancer subtypes [6,7]. As a consequence, inhibitors targeting altered cellular signal transduction in bone metastases are the subject of current research [8]. Therefore, bone-seeking sublines such as the MDA-MB-231 BO (231-BO) of the triple negative breast cancer cell line MDA-MB-231 were established using repetitive in vivo selection and were used to examine the molecular mechanisms and druggable targets of bone metastasis in several studies [9].

Generally, the formation of metastases in breast cancer is a multistep process including invasion at the primary site, intravasation, homing, adherence, extravasation, and colonization at the distant site like bone [3,10,11]. Therefore, it is not surprising that a large number of proteins regulate and facilitate this complex process of metastasis. Among them are, for example, receptor tyrosine kinases such as the ErbB2 receptor tyrosine kinase 2 (HER2) or the discoidin domain receptors 1 and 2 (DDR1/2) [12,13,14]. These proteins are often overexpressed in breast cancer and are associated with the occurrence of metastases in general [12,13,14,15].

Additionally, breast cancer cells with certain properties are more likely to metastasize to bone than others as an expression of the seed-and-soil theory by Paget [16,17,18]. Several studies identified factors that are overexpressed in bone-homing breast cancer cells and therefore, predict bone metastasis. These include interleukin 11 (IL11), connective tissue growth factor (CTGF), C-X-C chemokine receptor type 4 (CXCR4), matrix metalloproteinase 1 (MMP1), and others [19,20,21]. After homing to the bone marrow, breast cancer cells interact with bone cells such as osteoclasts and osteoblasts within a vicious cycle of osteolytic bone metastases. Breast tumor cells secrete factors such as CTGF, parathyroid hormone-related protein (PTHrP), IL11, and receptor activator of nuclear factor kappa-Β ligand (RANKL), which stimulate osteoclasts and osteoclastogenesis in part via stimulation of osteoblasts and thus, promoting bone resorption. Tumor cells, in turn, are stimulated by factors such as the transforming growth factor β (TGFβ), the insulin-like growth factor 1 (IGF1), and RANKL promoting cell proliferation, survival, and migration as well as further secretion of the above mentioned pro-osteolytic proteins. These growth factors are released from bone matrix during osteolysis or are secreted from bone cells like osteoblasts [3,8,17,19,20,21,22,23,24]. The polypeptide growth factor TGFβ constitutes the second most abundant factor stored in the bone matrix and therefore, is released during osteolysis [3]. TGFβ binds to its corresponding receptor on breast tumor cells and increases, for example, the expression of CTGF, which is a matricellular protein belonging to the CCN family. CTGF promotes RANKL-induced osteoclastogenesis and differentiation of osteoblasts [25,26].

The serine/threonine kinase AKT, also known as protein kinase B, is supposed to have an important role for bone metastasis of breast cancer [27,28]; the AKT signaling is frequently dysregulated in triple negative breast cancer among plenty of other important signaling pathways [29,30]. Additionally, former studies revealed that AKT in general suppresses apoptosis and promotes migration, invasion as well as metastasis in breast cancer [31,32,33,34]. More specifically, AKT regulates a plethora of the hallmarks of cancer such as survival and metabolism via several signaling pathways. For instance, AKT regulates the metabolism of cancer cells partly via inhibitory phosphorylation of its substrate glycogen synthase kinase 3 (GSK3) or modulates cell survival via inhibitory phosphorylation of the pro-apoptotic BAD protein [35]. In line with its role in bone metastasis, the expression of dominant negative AKT reduces bone metastasis of MDA-MB-231 cells in a mouse model [36]. More particularly, AKT consists of the three isoforms AKT1, AKT2, and AKT3 [37]. Despite their high similarity of about 80% at the amino acid level [33,38], the three AKT isoforms exert non-redundant and even opposing roles. Initial evidence for isoform-specific effects was generated in *Akt* isoform knockout mice. Knockout of *Akt1* impairs growth of mice, whereas knockout of *Akt2* leads to a diabetes-like phenotype and an *Akt3* knockout in mice reduces brain size [39,40,41]. In breast cancer, the AKT isoforms also show an inverse correlation in their function on tumor growth and metastasis, as previously reviewed [37]. On the one hand, AKT1 is mainly involved in the promotion of tumor growth through regulation of cyclin D1, retinoblastoma protein (Rb), and p21 [42,43,44,45]. On the other hand, AKT1 was shown to inhibit migration and metastasis by regulating integrin β1, MMP9, tuberous sclerosis complex 2 (TSC2), and palladin [42,44,46,47]. In opposition, AKT2 has been shown to promote metastasis by enhancing migration and invasion of breast cancer cells mediated by a regulation of F-Actin, vimentin, and integrin β1 [44,48,49]. However, investigations about the role of AKT3 in breast cancer are scarce and several laboratories reported only minor effects of AKT3 on tumor growth and metastasis [43]. Recently, an anti-migratory role of AKT3 was reported in the TNBC cell line MDA-MB-231 [50]. A knockdown of AKT3 results in an increased level of S100A4, which supports the formation of lung metastasis [51]. Although the AKT signaling pathway is hyperactive in bone-metastasizing breast cancer [27,52], the isoform-specific effect of AKT on the formation of breast cancer bone metastases and on the vicious cycle of osteolysis remains unrevealed. Therefore, we aimed to clarify the role of AKT3 in bone metastasis of breast cancer and provide a rationale for an isoform-specific AKT inhibition in breast cancer patients with bone metastases.

We observed a high level of pAKT in the bone-seeking breast cancer subline MDA-MB-231 BO. PanAKT inhibition, especially in combination with mTOR inhibition, led to reduced proliferation and migration. In more precise terms, isoform-specific kinase assay revealed an elevated activity of AKT3, but not AKT1 or AKT2, in 231-BO cells. For further investigations of AKT isoform-specific effects in bone-seeking breast cancer cells, we generated shRNA knockdowns of the AKT isoforms. Knockdown of AKT3 in 231-BO cells increased migration, invasion, and chemotaxis towards EGF as well as phosphorylation and signaling of metastasis-associated proteins HER2 and DDR1/2. Interestingly, knockdown of AKT3 resulted in a diminished increase in tumor-osteolysis associated CTGF expression after TGFβ-stimulation. In a xenograft intracardiac mouse model, AKT3 knockdown in 231-BO cells led to significantly higher metastasis to bone. Despite the increased metastatic burden in bones, the rate of osteolysis in the vertebral bodies was not elevated after injection of 231-BO AKT3 knockdown cells. Taken together, our data suggest that an AKT3 knockdown in the bone-seeking breast cancer cell line 231-BO increases metastasis to bone but does not facilitate the vicious cycle of osteolysis.

## 2. Materials and Methods

### 2.1. Chemicals and Reagents

Antibodies against AKT1, AKT2, AKT3, panAKT, pAKT S473, pAKT T308, pAKT1 S473, pAKT2 S474, pS6 S240/244, pGSK3α/β S21/9, pHER2 Y877, S100A4, MMP2, RANK, and CTGF, and secondary HRP-linked antibodies against rabbit or mouse IgG were purchased from Cell Signaling Technology Inc. (Danvers, MA, USA). Antibody against AKT3 was provided by Millipore (Burlington, MA, USA). Antibody against HSC-70 was purchased from Santa Cruz Biotechnology Inc. (Dallas, TX, USA). Antibody against pDDR1/2 Y796/Y740 was provided by R&D systems Inc. (Minneapolis, MN, USA). RAD001 was provided by Selleck Chemicals (Houston, TX, USA) and MK-2206 was obtained from AbMole BioScience Inc. (Houston, TX, USA). Recombinant human TGF-β1 and recombinant human EGF were purchased from PeproTech Inc. (Rocky Hill, NJ, USA).

### 2.2. Cell Culture

MDA-MB-231 parental cells were obtained from the American Type and Culture Collection (Rockville, MD, USA). The bone-seeking subline MDA-MB-231 BO has been described previously [9]. All cell lines were maintained in DMEM with 10% (*v*/*v*) FCS, and 1% (*v*/*v*) penicillin and streptomycin and cultured at 37 °C in a humidified atmosphere with 5% CO_2_. All cell lines were authenticated by multiplexion (Heidelberg, Germany) and mycoplasma-free status was confirmed. Cells were used at low passage numbers below 25 passages.

### 2.3. Lentiviral Knockdown

pLKO.1-puro vector encoding either scrambled shRNA or shRNAs directed against AKT1, AKT2, or AKT3 were obtained from Sigma-Aldrich (St. Louis, MO, USA). Pseudotyped lentiviral viruses were generated using transient lipofectamine-mediated cotransfection of HEK293T cells with the following plasmids: pLKO.1-puro (2.5 μg), pMD2.G-VSV-G (1 μg), and psPAX2-Gag-Pol (8 μg). Virus-containing supernatants were collected after 24 and 48 h and filtered. MDA-MB-231 BO cells were incubated with the respective virus supernatants and transduced cells were selected with culture medium containing 1.5 μg/mL puromycin (Sigma-Aldrich, St. Louis, MO, USA) for at least one week before experiments were performed.

### 2.4. Inhibitor Testing and Cell Viability Assay

Cells were seeded in a 96-well plate at a density of 1500 cells/well, allowed to adhere overnight, and were treated with various concentrations of MK2206 and RAD001 either alone or in combination for 72 h. Cell viability was measured using Alamar Blue Assay. Therefore, cells were incubated with 5 ng/mL resazurin (Sigma-Aldrich, St. Louis, MO, USA) for 4h and fluorescence-based absorption was detected at 540nm with a Tecan microplate reader (Tecan, Maennedorf, Switzerland). The effect of MK2206 and RAD001 on phosphorylation of AKT and S6 was confirmed using Western blot analysis of whole cell lysates after treatment with MK2006 and RAD001 either alone or in combination in various concentrations for 24 h.

### 2.5. Proliferation Assay

One thousand cells per well were seeded in a 96-well plate (Greiner Bio-One, Kremsmünster, Austria) and were allowed to adhere. The cells were incubated for the indicated time at 37 °C in a humidified atmosphere with 5% CO_2_. The live cell imaging system IncuCyte Zoom (Essen Bioscience, Ann Arbor, MI, USA) was used to automatically take phase contrast pictures every 2 h. Pictures were analyzed by creating a confluence mask with IncuCyte Zoom software (Essen Bioscience, Ann Arbor, MI, USA).

### 2.6. Migration and Invasion Assay

In a 96-well ImageLock plate (Essen Bioscience, Ann Arbor, MI, USA), 30,000 cells/well were seeded and were allowed to adhere to a nearly confluent monolayer. For the migration assay, the 96-well plate was coated with Matrigel (Corning Inc., New York, NY, USA) before cells were seeded. Scratch wounds were created according to the manufacturer’s instructions using the IncuCyte WoundMaker (Essen Bioscience, Ann Arbor, MI, USA). Wells were filled either with medium alone, with medium containing MK2206 and/or RAD001 or for the invasion assay, with medium containing Matrigel at a concentration of 8 mg/mL. Phase contrast pictures were taken automatically every 2 h by the IncuCyte Zoom live cell imaging system (Essen Bioscience, Ann Arbor, MI, USA). Wound closure was analyzed by creating a confluence and scratch mask with IncuCyte Zoom software (Essen Bioscience, Ann Arbor, MI, USA). Relative Wound Density was calculated automatically by the following equation:

$$\%\mathrm{RWD}(t)\;=100\cdot \;\frac{\left(w\left(t\right)\;-\;w\left(0\right)\right)}{\left(c\left(t\right)\;-\;w\left(0\right)\right)}$$

*w*(*t*) = density of wound region at time (*t*); *c*(*t*) = density of cell region at time (*t*).

### 2.7. Chemotaxis Assay

One thousand cells per well were seeded into the top insert of a 96-well ClearView plate (Essen Bioscience, Ann Arbor, MI, USA) and were allowed to settle down. EGF as a chemoattractant was added in the reservoir plate at a final concentration of 10 ng/mL. The insert was transferred to the reservoir plate and migration of cells through the pores of the 96-well ClearView plate was monitored by automatically taking pictures every 2 h with the IncuCyte Zoom live cell imaging system (Essen Bioscience, Ann Arbor, MI, USA). Chemotaxis to EGF was analyzed by measuring the confluence in the reservoir plate and compared with the respective control group without a chemoattractant gradient.

### 2.8. Western Blot

Cells were lysed either with NP40 lysis buffer (50 mM HEPES pH 7.5, 150 mM NaCl, 1% NP-40, 2% Aprotinin, 2 mM EDTA, 50 mM NaF, 10 mM NaPPi, 10% Glycin, 1 mM vanadate, and 1 mM PMSF), with M-PER lysis buffer (ThermoFisher Scientific Inc., Waltham, MA, USA) including phosphatase and protease inhibitor (Pierce Biotechnology Inc., Rockford, IL, USA), or with TCA lysis buffer. Proteins were separated by SDS–polyacrylamide gel electrophoresis and transferred to nitrocellulose paper. After incubation with the primary antibody, the membrane was incubated with anti-mouse IgG HRP-conjugated or anti-rabbit IgG HRP-conjugated secondary antibodies (Cell Signaling Technology Inc., Danvers, MA, USA). Protein levels were quantified using LAS-4000 Imager and AIDA Image Analyzer software v.3.44 (Elysia-Raytest, Angleur, Belgium). Ponceau staining was used to normalize the total amount of protein of each group.

### 2.9. AKT Isoform-Specific In Vitro Kinase Assay

Immunoprecipitation of AKT isoforms was performed by incubating whole cell lysates with AKT isoform-specific antibodies overnight at 4 °C to precipitate AKT isoforms after prior coupling of 2.5 μg of the corresponding antibody for 2h, rotating at 4 °C to 40 μL of equilibrated 50:50 slurry of protein G-sepharose (GE Healthcare, Chicago, IL, USA). Afterwards, beads were washed two times with NP40 lysis and two times with kinase buffer (Cell Signalling Technology Inc., Danvers, MA, USA) and incubated with 1 μg GSK3α/β fusion protein (Cell Signalling Technology Inc., Danvers, MA, USA) and 0.2 mM ATP (Cell Signalling Technology Inc., Danvers, MA, USA) for 30 min at 30 °C. Samples were then analyzed by Western blot technique as described above and probed with antibodies directed against pGSK3α/β (S9/21), panAKT, and IgG. Subsequently, the nitrocellulose membrane was incubated with secondary anti-mouse antibody to detect mouse IgG levels for sample correction.

### 2.10. Functional Kinome Profiling

Functional kinome profiling was used to analyze the activity of protein tyrosine kinases (PTK). Therefore, whole cell lysates were generated using M-PER Mammalian Extraction Buffer (ThermoFisher Scientific Inc., Waltham, MA, USA) with Halt Phosphatase Inhibitor and EDTA-free Halt Protease Inhibitor Cocktail (Pierce Biotechnology Inc., Rockford, IL, USA). The profiling was performed using the PamStation^®^12 (located at the UCCH Kinomics Core Facility, UKE, Hamburg, Germany) and corresponding PTK microarrays (PamChip^®^4) according to the manufacturer’s protocols (PamGene International, ‘s-Hertogenbosch, The Netherlands). A microarray contains 196 immobilized peptide sequences with 13 amino acids per sequence representing kinase-specific phosphorylation sites. Lysate containing 5 µg of protein and 400 µM ATP was applied per array. Phosphorylation of the kinase-specific peptide sequences was detected using fluorescein-labelled anti-phosphotyrosine antibodies (PamGene International, ‘s-Hertogenbosch, The Netherlands) and a CCD camera. Analysis of the intensity was conducted with the Evolve software v. 1.0 (PamGene International, ‘s-Hertogenbosch, The Netherlands) and for further analysis, the intensities were log2-transformed and proceeded with the BioNavigator software v. 6.0 (BN6, PamGene International, ‘s-Hertogenbosch, The Netherlands).

### 2.11. Intracardiac Mouse Model and Luciferase In Vivo Analysis

This experiment was carried out in strict accordance with the Guide for Care and Use of Laboratory Animals provided by the National Institutes of Health. All experimental protocols were approved by local authorities (Ministry of Health and Consumer Protection, Hamburg, Germany, Permit Number G52/11; N106/2018). Cells were transduced with virus-containing supernatant with the LeGO-iNeo2-Luc2 Luciferase-vector, provided by K. Rieken at the Research Department of Cell and Gene Therapy, UKE, Hamburg, Germany. Cells were selected with culture medium containing 1000 μg/mL Neomycin G418 (Thermo Fischer Scientific Inc., Waltham, MA, USA) for at least 14 days before injection. Then, 1 × 10^5^ cells in PBS were injected under general anesthesia in the left ventricle of female NSG mice at an age of eight weeks (obtained from Charles River Laboratories, Wilmington, MA, USA). Metastatic burden was monitored weekly by bioluminescence measurement with an IVIS imaging system (PerkinElmer Inc., Waltham, MA, USA) after intraperitoneal injection of Luciferin under general anesthesia. Mice were withdrawn from the experiment, if an abortion criterion was fulfilled (impaired movement of limbs or motor function, loss of body weight >20%, poor general condition) or after unsuccessful inoculation into the left ventricle indicated by either absence of bioluminescence signals in the whole mice or bioluminescence signals limited only to lungs. Upon necropsy after three weeks, ex vivo bioluminescence measurements of the whole mice, skeletal parts, and organs were performed. Bioluminescence data were analyzed using Living Image software (PerkinElmer Inc., Waltham, MA, USA). Radiographic analysis of the skeletal system ex vivo was conducted using X-ray images. Lower limbs and spine were fixed in 4% formalin. Lower limbs were decalcified, embedded in paraffin, and slices were HE-stained. The lumbar vertebral bodies L1 to L5 were embedded in methylmetacrylate and slices were stained with von Kossa/van Gieson staining, as described previously [53].

### 2.12. Statistical Analysis

Data were analyzed using GraphPad Prism 8 (GraphPad Software Inc., San Diego, CA, USA). Combination indices (CI) for RAD001 and MK2206 combinatorial treatment were calculated with the CompuSyn software [54,55]. CI values <0.1 were grouped as “very strong synergism” (+++++) and CI values of 0.1–0.3 were grouped as “strong synergism” (++++) [56]. Outliers were determined using Grubbs’ test with an alpha of 0.05 where necessary in the in vivo experiments. Statistical significance was tested using one-way ANOVA with Dunnett’s (for comparison with one group) or Tukey’s post hoc test (for comparison with all groups) when comparing more than two groups or using unpaired two-tailed Student’s t-test or one-sample t-test with a hypothetical value of 1 when comparing two groups. Results were considered significant if *p* < 0.05. Each experiment was performed at least in triplicate. All figures show mean values and error bars represent SD, unless indicated otherwise.

## 3. Results

### 3.1. The Bone-Seeking Subline MDA-MB-231 BO Exhibits Constitutively Elevated pAKT Levels and Is Responsive for panAKT and mTOR Inhibition

**Figure 1:**
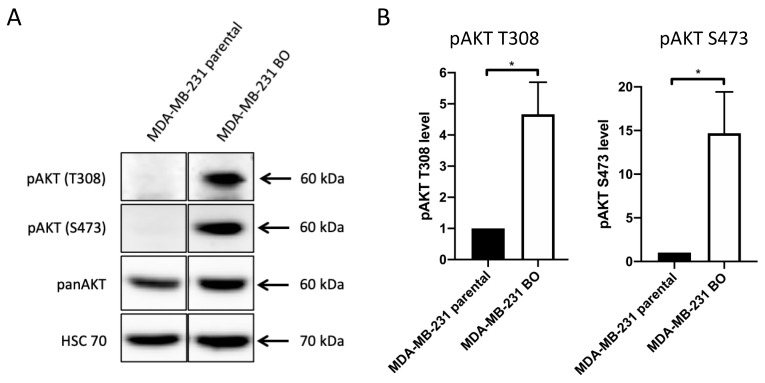
The bone-seeking subline MDA-MB-231 BO exhibits constitutively elevated pAKT levels compared to MDA-MB-231 parental cells: (**A**) Western blot assay was performed to determine pAKT T308, pAKT S473 and panAKT levels using antibodies directed against the indicated proteins. HSC70 functions as a loading control. (**B**) Relative levels of pAKT T308 and pAKT S473 in the bone-seeking MDA-MB-231 subline compared to MDA-MB-231 parental cells were quantified from Western blot analysis of triplicates. Data were normalized to Ponceau staining and panAKT level. Bars indicate mean with SD; * = *p* < 0.05.

Highly metastatic triple negative MDA-MB-231 cells form metastases in bones as well as lungs, liver, ovaries, and brain in a mouse model [57] (data not shown). The bone-seeking subline MDA-MB-231 BO (hereafter, 231-BO) was generated previously using repeated intracardiac inoculation of isolated bone metastatic MDA-MB-231 cells nine times [9]. This bone-seeking breast cancer cell line shows a predilection for formation of bone metastases and a reduced dissemination to lungs or other organs compared to the parental MDA-MB-231 cell line [9] (data not shown). To investigate the role of AKT in bone metastatic breast cancer cells, we performed Western blot analysis of pAKT T308 and S473 levels in the bone-seeking cell lines. The 231-BO cells displayed a 4.7-fold higher phosphorylation of AKT at T308 and a 14.7-fold higher phosphorylation of AKT at S473 normalized to panAKT expression in comparison to the MDA-MB-231 parental cell line (Figure 1A,B). There was no significant difference in the panAKT expression among the two cell lines. These data indicate a constitutive phosphorylation and thus, constitutive activation of AKT in the examined bone-seeking breast cancer cell line.

**Figure 2:**
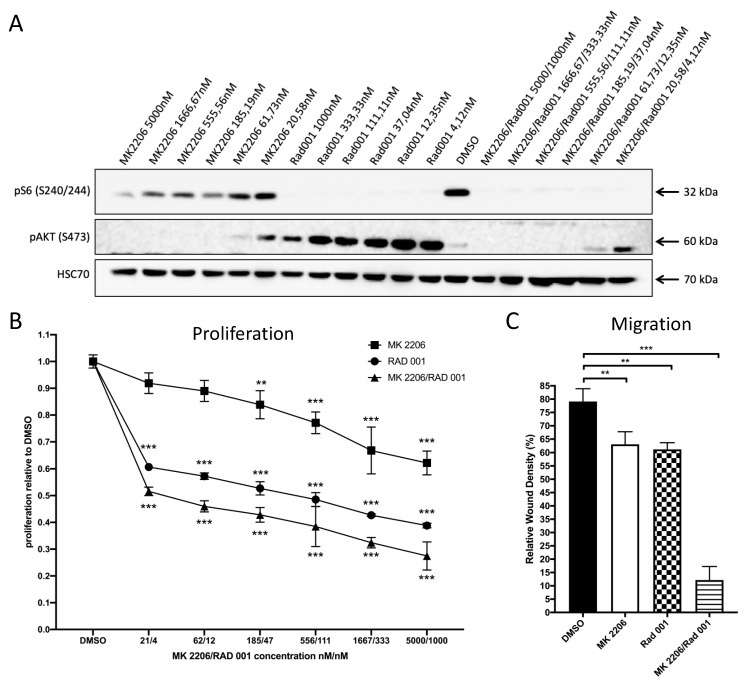
Treatment of 231-BO cells with the panAKT inhibitor MK2206 and the mTOR inhibitor RAD001 either alone or in combination inhibits proliferation and migration: (**A**) 231-BO cells were treated with MK2206 and RAD001 either alone or in combination with various concentrations for 24 h before preparing whole cell lysates. Inhibition of AKT (pAKT S473) and mTOR (pS6 S240/244) were confirmed by Western blot analysis using antibodies directed against the indicated proteins. HSC70 functions as a loading control. (**B**) 231-BO cells were seeded into a 96-well plate and were treated with various concentrations of MK2206 and RAD001 either alone or in combination for 72 h. Control cells were treated with DMSO. Cell viability was analyzed in triplicate using Alamar blue assay. Data points represent mean with SD; ** = *p* < 0.01; *** = *p* < 0.001. (**C**) Migration of 231-BO cells treated with MK2206 5000nM and RAD001 1000nM either alone or in combination was tested by scratch wound healing assay. Relative wound density was analyzed using the IncuCyte live cell imaging system. Bars indicate mean of triplicates with SD; ** = *p* < 0.01; *** = *p* < 0.001.

Prompted by these results, we sought evidence for the response of 231-BO cells to AKT inhibition and mTOR inhibition either alone or in combination using the allosteric panAKT inhibitor MK2206 and the mTOR inhibitor RAD001. Western blot analysis of 231-BO cells treated with MK2206 and/or RAD001 in various concentrations for 24 h revealed a strong reduction in pAKT S473 levels after MK2206 treatment and a marked decrease in pS6 S240/244 levels as an indicator of mTOR activity after treatment with RAD001 (Figure 2A). pAKT S473 levels were higher after single treatment with RAD001 and even in combination with low concentrations of MK2206 compared to DMSO control.

Following this, we examined the effect of MK2206 and RAD001 alone or in combination on the proliferation of 231-BO cells using an Alamar blue cell viability assay. Treatment of 231-BO cells with higher concentrations of MK2206 resulted in significant decreased proliferation with a calculated IC50 value of 15139.3 nM. However, treatment with MK2206 at lower concentrations (61.73 and 20.58 nM) was not sufficient to significantly reduce proliferation of 231-BO cells (Figure 2B). RAD001 significantly reduced the proliferation of 231-BO cells in all tested concentrations with a calculated IC50 value of 65.7 nM. Combinatorial treatment with MK2206 and RAD001 in a constant ratio of 5:1 resulted in a significant reduction in proliferation to a higher extent compared to treatment with MK2206 or RAD001 alone indicated by reduced IC50 values for MK2206 (37.8 nM) and RAD001 (7.6 nM) in combination.

Using the Chou and Talalay method to determine combination indices [54,55] revealed a strong to very strong synergistic effect of treatment with MK2206 and RAD001 in combination for the indicated concentrations (Figure S1).

Since AKT is known as a regulator of migration, we tested the effect of MK2206 and RAD001 either alone or in combination on the migration of 231-BO cells using scratch wound healing assays and live cell imaging. Treatment with MK2206 5000 nM or RAD001 1000 nM alone slightly decreased the migration of 231-BO cells, whereas combinatorial treatment with MK2206 and RAD001 5000/1000 nM showed a strong decrease in migration in comparison to DMSO control (Figure 2C).

Taken together, these data suggest a functional role of AKT/mTOR signaling for proliferation and migration in bone-metastatic 231-BO breast cancer cells.

### 3.2. Bone-Seeking 231-BO Cells Show an Increased AKT3 Activity in an In Vitro Kinase Assay

**Figure 3:**
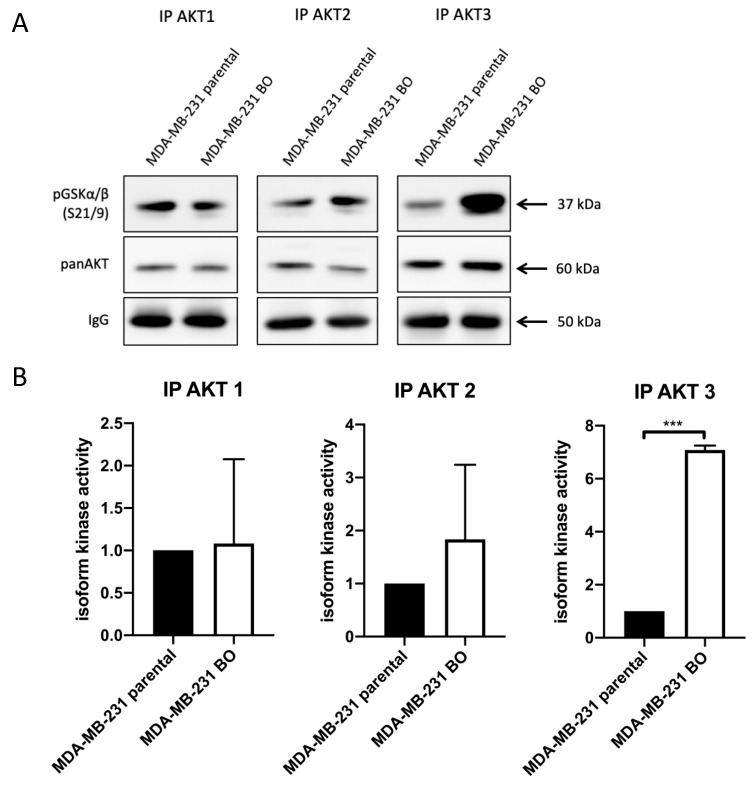
AKT3 activity is elevated in bone-seeking 231-BO cells in an in vitro kinase assay: (**A**) Isoform-specific in vitro AKT kinase assay was performed to determine differences in the bone-seeking subline 231-BO compared to parental MDA-MB-231 cells. After AKT isoform immunoprecipitation, GSK3α/β fusion protein was used as an AKT substrate. Level of pGSK3α/β S21/9 was detected as an indicator of AKT isoform activity. Antibodies directed against pGSK3α/β S21/9, panAKT, and mouse IgG were used after Western blotting. (**B**) Quantification of AKT isoform kinase activity indicated by phosphorylation of pGSK3α/β was performed from Western blot analysis of triplicates and normalized to intensity of IgG signals. Bars indicate mean relative to MDA-MB-231 parental cells with SD; *** = *p* < 0.001.

To further specify the elevated pAKT levels in the bone-seeking 231-BO cells, we performed an AKT isoform-specific in vitro kinase assay by immunoprecipitating the single AKT isoforms with antibodies against AKT1, AKT2, and AKT3 and incubation with GSK3α/β fusion protein as an AKT substrate. We revealed a 7.1-fold increase in AKT3 activity in the 231-BO cell line compared to parental MDA-MB-231 cells indicated by an elevated pGSK3α/β level (Figure 3A,B). There was no significant difference of AKT1 or AKT2 activity in 231-BO cells compared to the parental MDA-MB-231 cell line, though there was a slight but not significant increase in the AKT2 activity in the bone-seeking subline.

We confirmed the findings that AKT3 is the main isoform contributing to the enhanced AKT signaling in 231-BO cells by examining isoform-specific phosphorylation of AKT1 and AKT2. Therefore, we performed Western blot analysis with pAKT1 (S473) and pAKT2 (S474) antibodies. Unfortunately, no appropriate isoform-specific pAKT3 (S472) monoclonal antibody exists so far. Phosphorylation of AKT1 or AKT2 did not differ between 231-BO cells and the parental MDA-MB-231 cell line (Figure S2A). We concluded that if pAKT1 and pAKT2 are not elevated in the 231-BO cell line compared to the parental cell line, an enhanced phosphorylation of AKT3 might explain the higher pAKT level in general in the 231-BO cells.

### 3.3. Knockdown of AKT3 in Bone-Seeking 231-BO Cells Increases Migration, Invasion, and Chemotaxis towards EGF but Had No Effect on Proliferation

**Figure 4:**
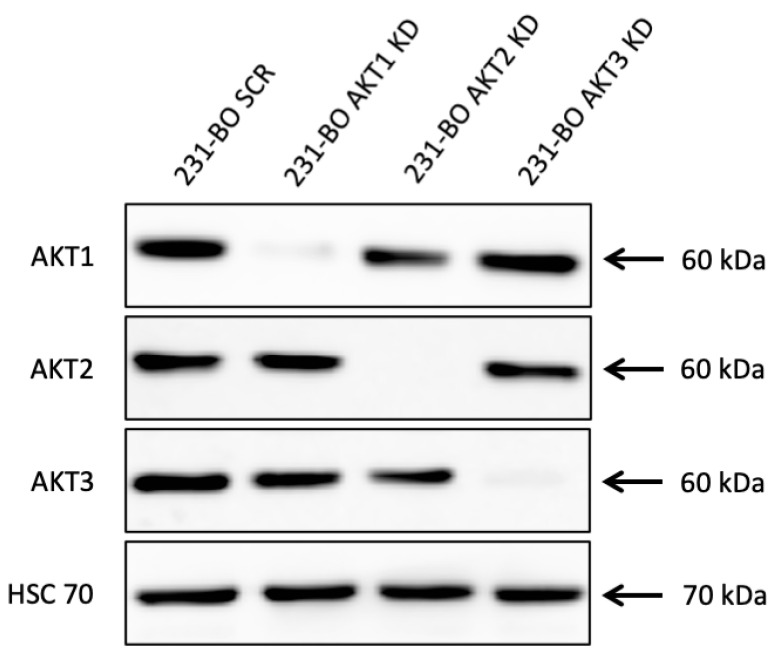
Lentiviral shRNA knockdown of AKT isoforms in 231-BO cells: Knockdowns of AKT isoforms were generated using lentiviral transduction of isoform-specific shRNA. Knockdown was confirmed by Western blot analysis and AKT1, AKT2, and AKT3 were detected with isoform-specific antibodies. HSC70 functions as a loading control.

Subsequently, we intended to investigate the effect of the single AKT isoforms on cellular mechanisms in the bone-seeking 231-BO cell line. Therefore, stable AKT isoform-specific knockdowns were generated on 231-BO cells by lentiviral transduction of shRNAs either against AKT1, AKT2, AKT3, or scrambled (SCR) control. Successful knockdown of AKT isoforms was confirmed by Western blot analysis, showing a knockdown of 92.9% for AKT1, of 97.7% for AKT2, and of 98.1% for AKT3 without affecting the expression of the non-targeted isoforms (Figure 4).

Again, we sought to confirm the findings from the in vitro AKT kinase assay by analyzing pAKT T308 and pAKT S473 levels in the 231-BO cells with AKT isoform knockdowns. The 231-BO cells with knockdown of AKT3 and to a lesser, non-significant extent also with knockdown of AKT2 exhibited lower pAKT T308 and pAKT S473 levels in comparison to SCR control (Figure S2B). Levels of pAKT T308 and of pAKT S473 in 231-BO cells with an AKT1 knockdown showed no significant difference compared to SCR control. This further supports our findings that AKT3 is the predominantly activated isoform in 231-BO cells.

**Figure 5:**
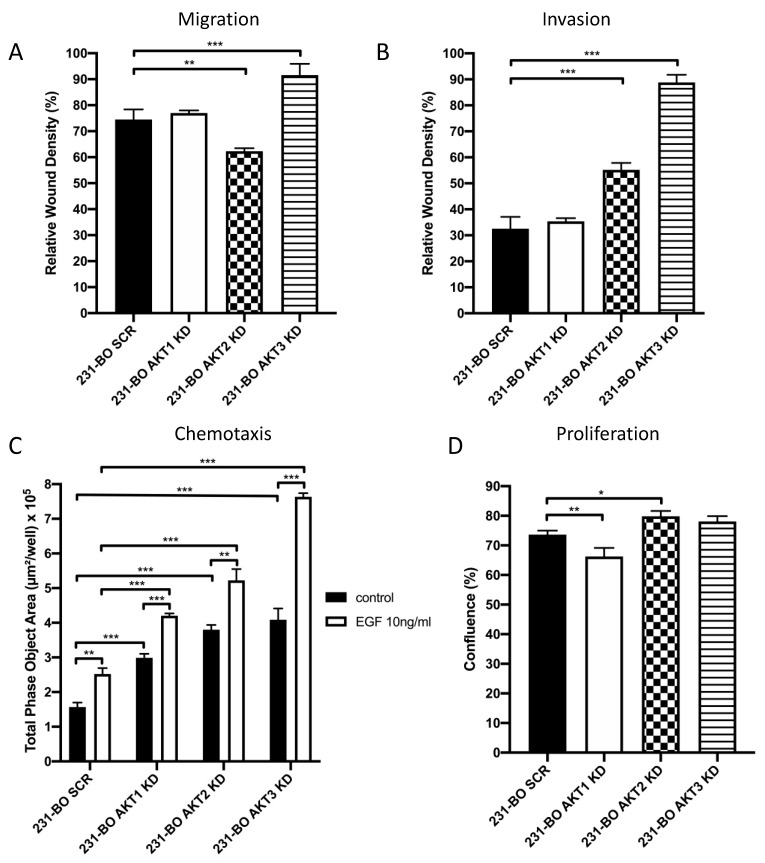
Knockdown of AKT3 in 231-BO cells promotes migration, invasion, and chemotaxis towards EGF but has no effect on proliferation: (**A**) Migration of 231-BO cells with AKT isoform knockdowns was tested using a wound healing scratch assay. Relative wound density was analyzed from triplicates by the IncuCyte live cell imaging system. Bars indicate mean with SD; ** = *p* < 0.01, *** = *p* < 0.001. (**B**) 231-BO AKT isoform knockdown cells were seeded in a 96 well plate in triplicate for invasion assay. Matrigel was added as an ECM after scratch wounds were created. The IncuCyte live cell imaging system was used to determine relative wound density. Bars indicate mean with SD; *** = *p* < 0.001. (**C**) Chemotaxis of 231-BO cells with AKT isoform knockdowns was tested with a Boyden chamber assay using the IncuCyte live cell imaging system. Cells were seeded in the upper chamber and EGF in a concentration of 10ng/mL was added to the lower chamber. Boyden chambers without an EGF gradient served as control. The area of the lower chamber occupied with transmigrated cells through pores was determined. Bars indicate mean with SD; ** = *p* < 0.01, *** = *p* < 0.001. (**D**) 231-BO cells harboring AKT isoform knockdowns were seeded in a 96-well plate and confluence was monitored with the IncuCyte live cell imaging system. Bars indicate mean with SD; * = *p* < 0.05, ** = *p* < 0.01.

Migration and invasion wound healing assays were performed by creating a scratch in a nearly confluent cell monolayer with or without adding Matrigel as an ECM-like matrix. Live cell imaging was conducted using the IncuCyte Zoom system. Knockdown of AKT3 significantly increased migration of 231-BO cells compared to SCR control (Figure 5A and Figure S3). Knockdown of AKT2 leads to a slight reduction in migration, whereas knockdown of AKT1 had no significant effect. Furthermore, we observed a marked increase in invasion in the 231-BO AKT3 knockdown cells in comparison to SCR control, whereas AKT1 knockdown again had no effect (Figure 5B and Figure S4). Surprisingly, knockdown of AKT2 also increases invasion of the 231-BO cell line. We could further show an elevated MMP2 expression in the AKT2 knockdown 231-BO cells compared to the SCR control (Figure S5).

To test the chemotaxis towards an EGF-gradient, we conducted Boyden chamber assays determining the transmigrated cells in the lower chamber with the live cell imaging IncuCyte Zoom system. Again, 231-BO cells lacking AKT3 exhibit an enhanced chemotaxis towards EGF compared to the SCR control (Figure 5C). Knockdown of AKT1 and AKT2 also displayed an increased chemotaxis towards EGF compared to the SCR control. Although all AKT isoform knockdowns showed an increased transmigration without an EGF gradient and an enhancement in transmigration after adding an EGF gradient, the 231-BO AKT3 knockdown cells exhibit the highest increase in chemotactic migration after exposition to an EGF gradient (1.87-fold increase vs. 1.61-fold increase in SCR control, 1.41-fold increase in AKT1 knockdown, and 1.37-fold increase in AKT2 knockdown).

We determined the proliferative behavior of 231-BO cells with AKT isoform knockdowns using the IncuCyte Zoom live cell imaging system. Knockdown of AKT3 did not display a significant alteration in proliferation compared to the SCR control, whereas knockdown of AKT1 slightly decreased proliferation and knockdown of AKT2 slightly increased proliferation of 231-BO cells (Figure 5D).

Our data suggest that AKT3 plays a crucial role in the steps of breast cancer metastasis as knockdown of AKT3 in bone-seeking breast cancer cells increases migration, invasion, and chemotaxis towards EGF without affecting cell proliferation.

### 3.4. Knockdown of AKT3 in 231-BO Cells Promotes Metastasis to Bone after Intracardiac Inoculation but Does Not Increase Osteolysis

**Figure 6:**
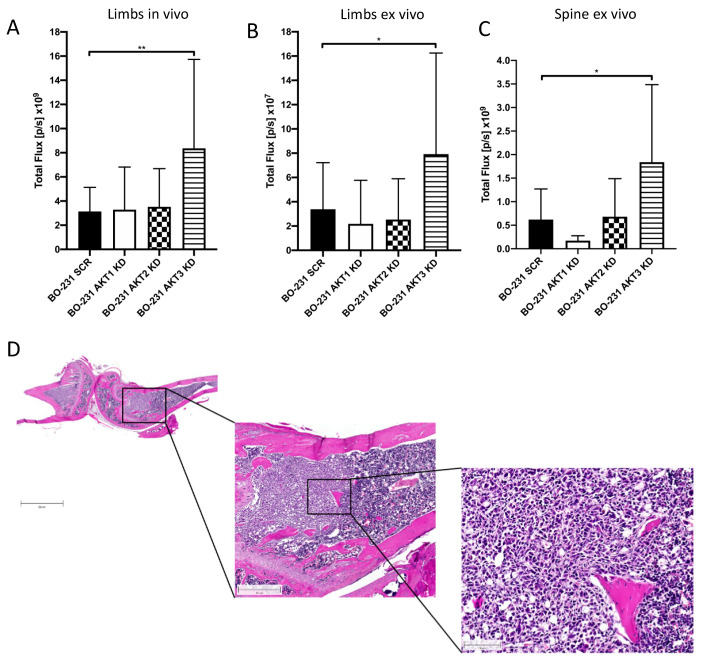
Knockdown of AKT3 in 231-BO cells shows increased metastasis to bone after intracardiac inoculation in NOD scid gamma (NSG) mice: To test the effect of AKT isoform knockdowns on metastasis in an in vivo model, 231-BO cells with AKT isoform knockdowns were transduced with a luciferase vector and were inoculated into the left ventricle of NSG mice. Correct injection, tumor cell dissemination, and growth were monitored by measurement of bioluminescence. Mice were sacrificed after 21 days, exenterated, and hind limbs as well as lumbar vertebrae were harvested. Bioluminescence intensities of hind limbs of living mice (SCR and AKT1 KD: *n* = 16; AKT2 KD and AKT3 KD *n* = 18) (**A**), of hind limbs ex vivo (SCR and AKT1 KD: *n* = 16; AKT2 KD and AKT3 KD *n* = 18) (**B**), and of the spine ex vivo (SCR and AKT1 KD: *n* = 8; AKT2 KD and AKT3 KD *n* = 9) (**C**) were measured by an IVIS imaging system after intraperitoneal luciferin injection. Bars indicate mean with SD; * = *p* < 0.05, ** = *p* < 0.01. (**D**) Right hind limbs were decalcified, embedded, and histological slices were generated. HE staining was used to confirm the presence of intraosseous tumor colonization. Image represents slices from a mouse injected with 231-BO AKT3 KD cells. Scale bars represent following dimensions: left image: 2000 μm; middle image: 500 μm; right image: 100 μm.

To further specify the role of AKT isoforms in metastasis of bone-seeking 231-BO cells, we utilized an in vivo mouse model. Luciferase transduced cells with AKT isoform knockdowns were inoculated into the left ventricle of NOD scid gamma mice (NSG mice) as an established model of bone metastasis [58] and formation of metastases was monitored by bioluminescence detection. Measurement of bioluminescence intensity in the hind limbs in vivo after 21 days and ex vivo as well as in the spine ex vivo revealed an increased bone metastasis in 231-BO cells lacking AKT3 compared to the SCR control, whereas knockdown of AKT1 or AKT2 had no effect on the formation of osseous metastases (Figure 6A–C and Figure S6). The presence of metastases in hind limbs was confirmed by histological slices of decalcified, HE-stained bones (Figure 6D).

Additionally, inoculation of 231-BO cells with an AKT1 knockdown led to a decreased formation of metastases in the adrenal glands compared to SCR control, whereas AKT2 or AKT3 knockdown had no significant effect (Figure S7). We could not observe any significant difference in metastasis to liver or ovary among the AKT isoform knockdowns (data not shown).

**Figure 7:**
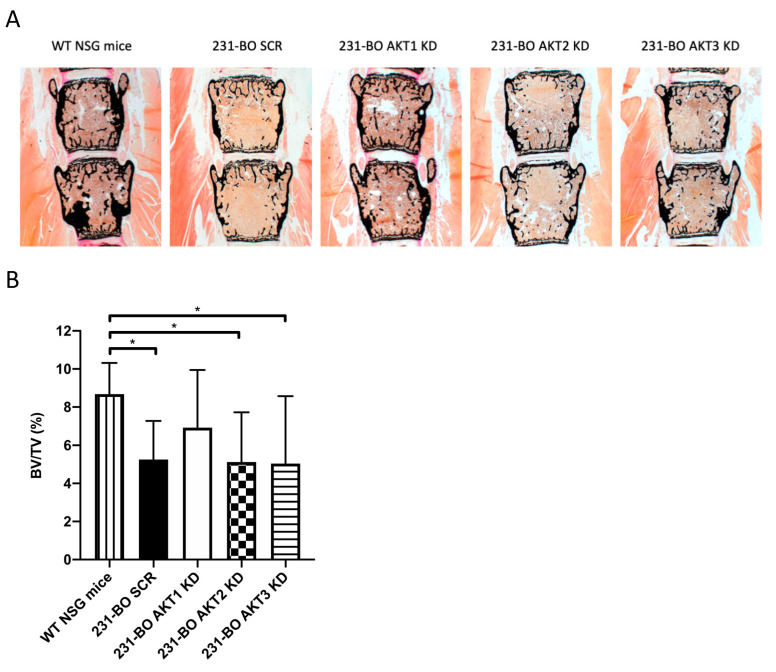
Knockdown of AKT3 in 231-BO cells does not enhance osteolysis in lumbar vertebral bodies after intracardiac injection: (**A**) Lumbar vertebrae bodies 1 to 5 were harvested from sacrificed mice after intracardiac injection of 231-BO cells with AKT isoform knockdowns and embedded in methylmetacrylate. Slices were stained with von Kossa/van Gieson staining to determine calcified bone volume. (**B**) Calcified bone volume was quantified relative to total tissue volume. WT NSG mice without tumor cell injection serve as a control (SCR, AKT1 KD, AKT2 KD, and AKT3 KD *n* = 25; WT NSG mice *n* = 6). Bars indicate mean with SD; * = *p* < 0.05.

To test the hypothesis that the enhanced metastatic burden in bones in the AKT3 knockdown of 231-BO cells was accompanied by an increased osteolysis, we performed von Kossa/van Gieson staining of slices from the lumbar vertebral bodies and analyzed the calcified bone volume in relation to the whole tissue volume (BV/TV). We observed a decrease in calcified bone volume after inoculation of 231-BO cells with AKT2 knockdown, AKT3 knockdown, and SCR control vector compared to NSG mice without tumor cell inoculation (Figure 7A,B). Surprisingly, AKT3 knockdown as well as knockdown of AKT1 or AKT2 did not affect the amount of calcified bone volume as an indicator for osteolysis compared to the SCR control. This stands in contrast to the higher metastatic burden in mice inoculated with AKT3 knockdown 231-BO cells. Accordingly, we could not observe any difference in the formation of osteolysis in radiographic images of the mice ossature (Figure S8).

Taken together, AKT3 knockdown in the 231-BO cell line promotes metastasis to bone after intracardiac inoculation into NSG mice but does not cause increased osteolysis in the spine.

### 3.5. 231-BO Cells with an AKT3 Knockdown Exhibit Elevated Activity and Phosphorylation of Metastasis-Associated HER2 and DDR1/2

**Figure 8:**
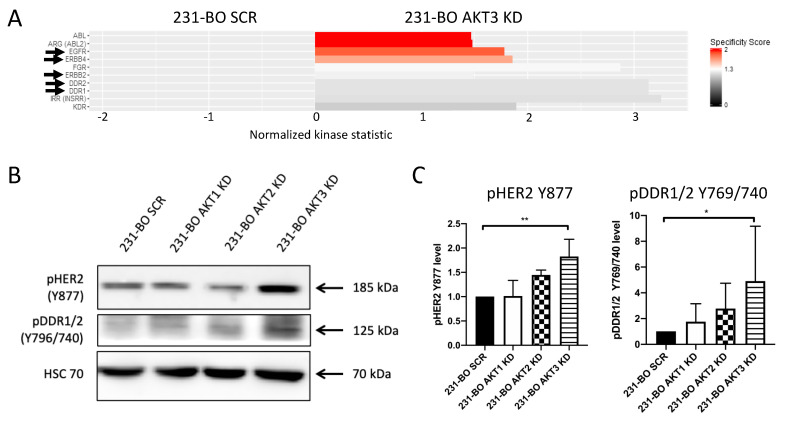
Knockdown of AKT3 in 231-BO cells increases activity and phosphorylation of HER2 and DDR1/2: (**A**) To identify alterations in tyrosine kinase activity in the AKT3 knockdown of 231-BO cells, we performed functional kinome profiling of whole cell lysates. The resulting upstream kinase analysis of 231-BO SCR versus 231-BO AKT3 KD is depicted (normalized kinase statistic (log2) > 0: higher kinase activity in KD; specificity score (log2) > 1.3; white to red bars: statistically significant changes). Arrows indicate kinases that are discussed in the following. (**B**) Western blot analysis was conducted as a proof of principle for some identified upregulated kinases from kinome profiling. Antibodies directed against the indicated proteins were used. HSC70 functions as a loading control. (**C**) Relative levels of pHER2 Y877 and pDDR1/2 Y769/740 in 231-BO AKT isoform knockdowns compared to 231-BO SCR cells were quantified from Western blot analysis of triplicates. Data were normalized to Ponceau staining. Bars indicate mean with SD; * = *p* < 0.05; ** = *p* < 0.01.

Consequently, we searched for alterations in signal transduction and kinase activities in 231-BO cells lacking AKT3 to disclose possible mechanisms that account for the promotion of migration and metastasis. Therefore, we screened the AKT3 knockdowns of the 231-BO cell line for upregulated kinase activities by performing functional kinome profiling using a PamStation^®^12. The resulting upstream kinase analysis predicts several kinases to be upregulated in the 231-BO AKT3 knockdown cells relative to SCR control (Figure 8A; red to light grey bars). Among the top 10 kinases, we identified the following kinases as possible mediators of enhanced migration and metastasis in AKT3 knockdown 231-BO cells: EGFR, ErbB2, ErbB4, DDR1, and DDR2. These kinases are associated with an enhanced migration, chemotaxis, and/or metastasis of breast cancer cells [12,13,59,60]. To confirm the results from kinome profiling, we analyzed the phosphorylation levels of ErbB2 (pHER2-Y877) and DDR1/2 (pDDR1/2-Y796/740) as a proof of principle by Western blotting of cell lysates from AKT isoform knockdowns of 231-BO cells. Cells with an AKT3 knockdown, but not with an AKT1 or AKT2 knockdown, showed a significantly increased phosphorylation of HER2 and DDR1/2 relative to SCR control (Figure 8B,C). Taken together, knockdown of AKT3 in bone-metastasizing 231-BO cells leads to elevated phosphorylation and activity of the pro-metastatic proteins HER2 and DDR1/2. EGFR and ErbB4 depict further kinases with an elevated activity in the AKT3 knockdown cells identified by kinome profiling.

Grottke et al. revealed an increased expression of S100A4 as an explanation for enhanced migration and metastasis to lung in an AKT3 knockdown of MDA-MB-231 parental cells [51]. We tested this hypothesis in our bone-seeking sublines and observed no detectable S100A4 expression in 231-BO (Figure S9). This suggests a minor role of S100A4 in bone-seeking sublines of the MDA-MB-231 cell line and, therefore, expression of S100A4 could not serve as an explanation of enhanced migration and metastasis in the AKT3 knockdown of 231-BO cells.

### 3.6. Knockdown of AKT3 in the 231-BO Cell Line Results in a Diminished Increase in the Vicious Cycle-Associated CTGF after TGFβ-Stimulation

**Figure 9:**
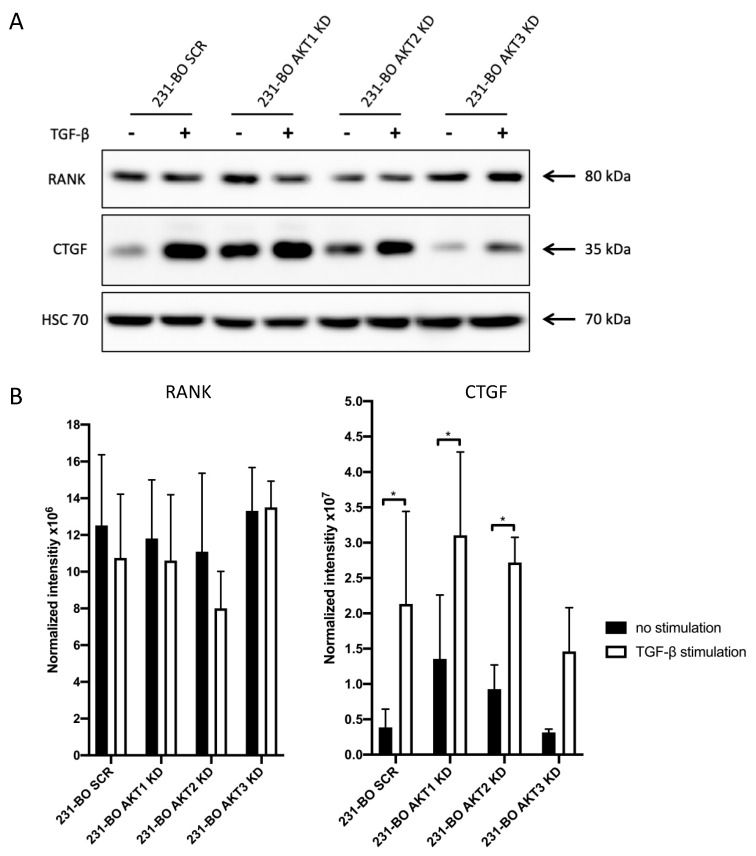
Knockdown of AKT3 in 231-BO cells results in a diminished increase in CTGF expression after TGFβ-stimulation: (**A**) 231-BO cells with AKT isoform knockdowns were stimulated with 5ng/mL TGFβ for 24 h prior to cell lysate preparation. Western blot analysis of vicious cycle associated proteins was performed using antibodies directed against the indicated proteins. HSC70 functions as a loading control. (**B**) Expression of CTGF and RANK indicated by the normalized intensity in the 231-BO AKT isoform knockdowns after TGFβ-stimulation or without stimulation was quantified from Western blot analysis in triplicate. Data were normalized to Ponceau staining. Bars indicate mean with SD; * = *p* < 0.05.

To understand why osteolysis in AKT3 knockdown 231-BO cells was not increased despite the elevated metastatic burden in bones, we analyzed CTGF and RANK expression after TGFβ-stimulation of 231-BO cells with AKT isoform knockdowns. CTGF and RANK are part of the vicious cycle in bone metastasis that promotes osteolysis [61,62]. We therefore tested the hypothesis that knockdown of AKT3 hampered CTGF and/or RANK expression in 231 BO cells. Indeed, we found that TGFβ-mediated upregulation of CTGF was significant in SCR, 231-BO AKT1 knockdown, and 231-BO AKT2 knockdown cells, whereas knockdown of AKT3 in 231-BO cells resulted in a diminished and not significant increase in CTGF expression after TGFβ-stimulation (Figure 9A,B). There was no significant difference in the expression of RANK after TGFβ-stimulation among the AKT isoform knockdowns in the 231-BO cells.

These results suggest an important role of AKT3 for TGFβ-stimulated CTGF expression in 231-BO cells.

## 4. Discussion

In this study, we provide evidence that AKT signaling has a crucial role in the metastasis of human breast cancer cells, especially to the bone in an isoform-specific manner in which AKT3 plays the leading role. To investigate the role of AKT in bone metastasis of breast cancer, we used a bone-seeking subline of the triple negative breast cancer cell line MDA-MB-231. The bone-seeking cell line 231-BO is well established and was generated by repeated in vivo passages in bone after intracardiac injection of MDA-MB-231 parental cells. The 231-BO cells are widely used as a model to investigate mechanisms and the treatment of bone-metastasizing breast cancer [9,52]. This cell line exhibits a more aggressive phenotype with increased proliferation, higher EMT-markers [52], impaired growth inhibitory effects of TGFβ, and promoted bone metastasis with larger osteolytic lesions [9]. Intracardiac inoculation of 231-BO cells results in a preferential metastasis to bone without metastasis to other organs and without affecting the tumorigenicity at the primary site, indicating its bone-specificity [9].

AKT signaling is frequently dysregulated in the triple negative breast cancer subtype, since AKT signaling is involved in the regulation of several cancer hallmarks such as survival, metabolisms, cell motility, and metastasis [29,35]. Several laboratories revealed an enhanced AKT signaling in bone metastatic breast cancer cells [27,28,36]. Furthermore, a microarray gene expression analysis of human breast cancer showed the PI3K/AKT signaling to be associated with bone metastasis [28]. We could also observe increased pAKT T308 and S473 levels in the bone-seeking subline 231-BO, corresponding to the results Fritsche et al. reported [52]. Therefore, our data suggest a constitutive phosphorylation and activation of AKT signaling in bone-seeking breast cancer cells. We speculated that this might lead to a response of the 231-BO cells to AKT inhibition. We tested this hypothesis by using the panAKT inhibitor MK2206 alone and in combination with the mTOR inhibitor RAD001. This combination is well-established since treatment with RAD001 alone was shown to activate AKT in a feedback loop lowering its therapeutical potential. Treatment with a combination of both drugs synergistically inhibits cancer cell proliferation [63,64,65]. We revealed here for the first time that the synergistic inhibition of proliferation as well as migration using a combined treatment with MK2206 and RAD001 can also be observed in bone-seeking breast cancer cells. The combination of both drugs could further increase the susceptibility of the single drugs indicated by reduced IC50 values in the combination therapy. We also observed the elevation of pAKT after RAD001 treatment using low concentrations of MK2206, suggesting that low concentrations of MK2206 were not sufficient to prevent this feedback loop of AKT activation. Higher concentrations of MK2206, in turn, were able to successfully inhibit this feedback activation of AKT.

The AKT family consists of the three isoforms AKT1, AKT2, and AKT3, which are shown to be partly inversely correlated in the regulation of breast cancer growth and metastasis [37]. Upregulation of AKT3 mRNA and amplification of *Akt3* are reported more frequently in triple negative breast cancer cells than in other breast cancer subtypes [50]. Furthermore, we specified the increased levels of pAKT T308 and S473 in the bone-seeking 231-BO cells as an elevated AKT3 kinase activity using an isoform-specific in vitro kinase assay. Western blot analysis of pAKT S473 and T308 in 231-BO cells with AKT isoform knockdowns revealed a diminished AKT phosphorylation in AKT3 knockdown 231-BO cells. Additionally, pAKT1 S473 and pAKT2 S474 did not differ between parental MDA-MB-231 and 231-BO cells, suggesting AKT3 is the leading cause for the elevated pAKT level in general in 231-BO cells. Unfortunately, no specific monoclonal pAKT3 S472 antibody exists so far to confirm an elevated phosphorylation of AKT3 in 231-BO cells. The findings further support our data from the in vitro kinase assay that AKT3 is the predominantly activated AKT isoform in 231-BO cells compared to the parental MDA-MB-231 cell line. This points to an important role of AKT3 in bone-metastasizing triple negative breast cancer cells that, to our knowledge, is reported here for the first time. To further investigate the functional role of AKT isoforms in bone-seeking breast cancer cells, we generated shRNA-mediated AKT isoform-specific knockdowns in the 231-BO cell line. We observed an increase in migration, invasion, and chemotaxis towards EGF in 231-BO cells with an AKT3 knockdown in vitro.

Chin et al. also observed an increase in migration but not invasion of triple negative breast cancer cells with an AKT3 knockdown. In contrast, knockdown of AKT3 leads to an impaired spheroid growth and tumor growth in vivo via an upregulation of p27 [50]. Knockdown of the AKT3 splice variant AKT3-S472 increases tumor growth in vivo through downregulation of Bim via an activation of the MAPK-signaling [66]. Chung et al. reported a decreased expression of AKT3 after overexpression of N-cadherin increases the motility of breast cancer cells [67]. An increase in migration was also observed in vascular tumor cells with an AKT3 knockdown [68]. Grottke et al. reported an enhanced migration and metastasis to lungs after knockdown of AKT3 in parental MDA-MB-231 cells via an upregulation of S100A4 [51]. In contrast, S100A4 expression was not detectable in the bone-seeking subline 231-BO in our experiments. This is in line with previous studies reporting a downregulation of S100A4 in other bone-seeking sublines of the MDA-MB-231 cell line [69,70]. We did not find an effect of an AKT3 knockdown on proliferation of 231-BO cells. Santi et al. reported a decrease in proliferation of triple negative breast cancer cells after knockdown of AKT3 [71], whereas Chung et al. did not observe an alteration of proliferation in the AKT3 knockdown, as we reported [67].

Additionally, we observed a decreased migration and an increased proliferation in the AKT2 knockdown of 231-BO cells. These results are in line with previous studies, reporting a pro-migratory but anti-proliferative effect of AKT2 [37,44,48,49]. Furthermore, we also confirmed the previously reported pro-proliferative role of AKT1 for breast cancer cells in the 231-BO cell line [37,42,44,45]. Surprisingly, we observed an enhanced invasive potential of 231-BO cells with a knockdown of AKT2 despite a suppressed migration. This could be explained by an increased MMP2 expression, which we observed in the AKT2 knockdown cells. MMP2 serves as a matrix-degrading enzyme and, therefore, could promote invasion but not migration [72].

As migration, invasion, and chemotaxis are steps of the metastatic cascade [10], we further questioned if the knockdown of AKT3 in 231-BO cells influences the metastatic potential in vivo. Therefore, we used a well-established model to investigate bone metastasis of breast cancer cells in mice by injecting 231-BO luciferase transduced cells with AKT isoform knockdowns into the left ventricle of the heart of immune-deficient NSG mice. We showed for the first time that inoculation of AKT3 knockdown 231-BO cells in NSG mice resulted in an enhanced metastasis to bone measured by bioluminescence imaging. The increase in bone metastasis after inoculation of 231-BO cells with an AKT3 knockdown is not due to an increased basic proliferation, since knockdown of AKT3 in 231-BO cells did not show an effect on proliferation in vitro.

Several studies investigated molecular mechanisms, which are responsible for an AKT isoform-specific signaling in breast cancer cells. For instance, Chin and Toker identified palladin as an actin-binding protein, which becomes phosphorylated on S507 and is activated specifically by AKT1. This isoform specificity is mediated by the linker region of AKT1 [47]. In glioblastoma, AKT2 and AKT3 but not AKT1 promote tumor progression, and alterations in the polarity of the PH domain and the regulatory domain were identified as responsible mechanisms for the isoform-specific effect [73].

**Figure 10:**
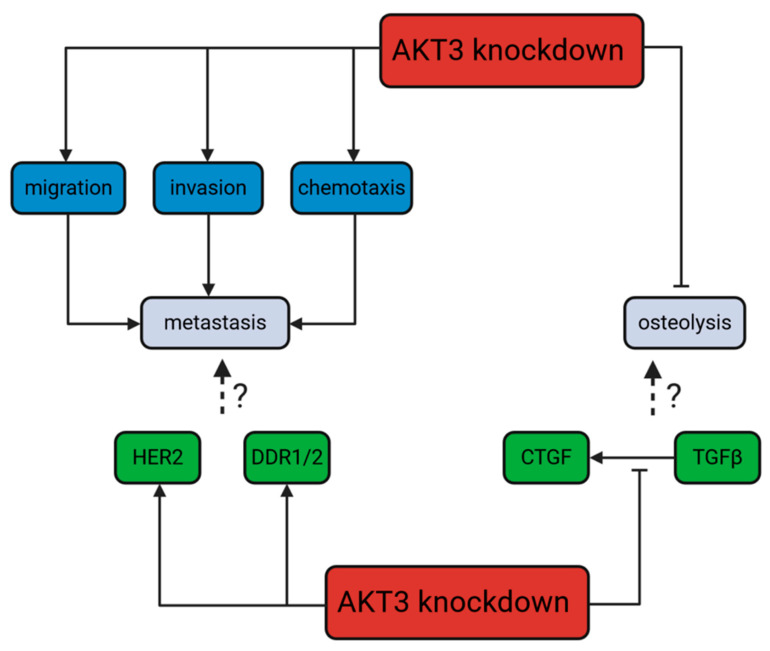
Dichotomic role of an AKT3 knockdown in 231-BO cells: promotion of metastasis but not osteolysis: On the one hand, knockdown of AKT3 increases, among others, DDR1/2 and HER2 signaling in bone-seeking breast cancer cells as well as promoting migration, invasion, and chemotaxis as steps of the metastatic process in general. HER2 and DDR1/2 activity is associated with metastasis in breast cancer in the literature. On the other hand, knockdown of AKT3 decreases the TGFβ-stimulated CTGF expression. In connection to these findings, knockdown of AKT3 leads to an absent promotion of osteolysis. The TGFβ/CTGF axis is correlated with the vicious cycle of osteolysis in breast cancer in the literature.

To investigate possible underlying molecular mechanisms of the increased metastasis to bone in the AKT3 knockdown 231-BO cells in our experiments, we performed tyrosine kinase profiling screening for upregulated kinase activities in the 231-BO cells lacking AKT3 compared to the SCR control. Among other kinases, we identified HER2, DDR1/2, and EGFR activities in the kinome profiling to be upregulated in AKT3 knockdown 231-BO cells. In addition, we confirmed these findings by reporting an enhanced phosphorylation of HER2 and DDR1/2 in Western blot analysis as a proof of principle for the kinome profiling data. This indicates an elevated HER2 and DDR1/2 and presumably EGFR signaling in 231-BO cells with an AKT3 knockdown (Figure 10).

HER2, DDR1/2, and EGFR are receptor tyrosine kinases which are frequently upregulated in breast cancer and are activated by various ligands e.g., heregulin, collagens, or EGF, respectively. Activated receptor tyrosine kinases mediate the extracellular signal via downstream signaling pathways such as PI3K/AKT or mitogen-activated protein kinase (MAPK) signaling, regulating several cellular processes [12,13,14,74]. EGFR signaling was shown to promote migration, invasion, and chemotaxis towards EGF and metastasis in breast cancer cells. Underlying molecular mechanisms are, for instance, the upregulation of MMP9, phosphoinositide phospholipase Cγ (PLCγ), and osteopontin [59,75,76,77]. The pro-metastatic effect of EGFR is further supported by studies reporting a correlation of EGFR and metastasis in human breast cancer probes [78]. Amplification of *Her2* is also correlated with a higher rate of breast cancer metastasis [12,13]. As possible mechanisms of the pro-migratory and pro-metastatic effect of HER2, higher levels of MMP2, MMP9, vascular endothelial growth factor (VEGF), zinc finger E-box-binding homeobox 1 (ZEB1), and lower levels of E-cadherin were reported [79,80,81]. Analysis of the preferential site of metastasis revealed that HER2-positive breast cancer cells preferably metastasize to brain, lung, and liver compared to HER2-negative cells [82,83,84]. Another study revealed that HER2 positive breast cancer shows a higher incidence of bone metastases than triple negative breast cancer [6]. Upregulation of HER2 activity in the AKT3 knockdown of 231-BO cells could mimic the bone-metastatic behavior of HER2 positive breast cancer and could explain the increase in bone metastasis. Upregulation of DDR1 promotes migration of breast cancer cells [60] and DDR1 and DDR2 are associated with metastatic breast cancer [14,15]. DDR1/2 could promote migration, invasion, and metastasis via an upregulation of MMPs, SNAIL1-mediated EMT, and protein kinase C (PKC)/Janus kinase 2 (JAK2)/signal transducer and activator of transcription 3 (STAT3) axis-mediated sex-determining region Y box 2 (SOX2) and NANOG expression [15,85,86]. An involvement of DDR1/2 in bone metastasis can be assumed, because DDR2 activates the runt-related transcription factor 2 (RUNX2), which is involved in osteoblast differentiation [15,61].

To our knowledge, this is the first time that isoform-specific knockdown of AKT3 as a downstream substrate of HER2 and DDR1/2 was reported to enhance activation of these proteins. However, the mechanisms by which a knockdown of AKT3 enhances signaling of receptor tyrosine kinases like HER2 or DDR1/2 remain unclear. A few data reported an upregulation of HER3 and EGFR phosphorylation after panAKT inhibition. According to Chandarlapaty et al., this can be explained due to an mTORC1 inhibition-mediated phosphorylation of HER3 and a forkhead box O (FOXO)-dependent upregulation of HER3 expression after AKT inhibition [87,88]. Inhibition of panAKT in mammary epithelial cells suppresses PIKfyve-dependent EGFR degradation and, therefore, induces a prolonged and increased EGFR signaling as a feedback loop mechanism. This mechanism for regulation of degradation through AKT was also found for further receptor tyrosine kinases [89]. Furthermore, an upregulation of DDR1 or DDR2 through other receptor tyrosine kinases was reported. IGF1, for example, induces the expression of DDR1 in breast cancer cells [60]. On that basis, expression and activation of DDRs could be upregulated via the enhanced signaling of other receptor tyrosine kinases in the AKT3 knockdown cells.

As HER2, DDR1/2, and EGFR are associated with metastasis of breast cancer, one could assume that the enhanced activity of these receptor tyrosine kinases mediates the enhanced migration, invasion, chemotaxis, and metastasis to bone in the AKT3 knockdown 231-BO cells. However, this hypothesis requires further experiments for confirmation.

Bone-metastasizing breast cancer cells interact with bone cells like osteoblasts and osteoclasts within a vicious cycle of osteolysis. Breast cancer cells are stimulated by factors such as TGFβ that are stored in the bone matrix and are released during bone resorption. Stimulated breast cancer cells in turn produce factors such as CTGF that directly or indirectly stimulate osteoclasts and, therefore, enhance osteolysis [3,20,21,23]. To investigate the role of AKT isoforms in tumor-induced osteolysis, we analyzed the formation of osteolysis in vertebral bodies after intracardiac inoculation of the 231-BO AKT isoform knockdown cells. We revealed an absent increase in osteolysis in the AKT3 knockdown of 231-BO cells despite the about 3-fold higher tumor burden. A higher tumor burden and, therefore, elevated number of tumor cells is supposed to enhance the stimulation of osteoclasts according to the vicious cycle model of osteolysis and thus, is supposed to promote the formation of osteolytic lesions. We concluded that the absent increase in osteolysis is an expression of a diminished or altered vicious cycle. To test this hypothesis, we stimulated 231-BO AKT isoform knockdown cells with TGFβ and measured the expression of the two vicious cycle proteins RANK and CTGF. There was no significant alteration in the expression of RANK among the AKT isoform knockdowns, whereas knockdown of AKT3 was shown to diminish the increase in CTGF after TGFβ-stimulation. Since CTGF is a driver of osteoclast differentiation and osteolysis within the vicious cycle, this could be an explanation for the absent increase in osteolysis in the bone metastases of AKT3 knockdown 231-BO cells. Moreover, 231-BO cells were harvested from already formed bone metastases and thus, may represent a later stage in bone metastasis in which an activity signature that promotes the vicious cycle plays a crucial role. This could be an explanation for the elevated AKT3 activity in the 231-BO cells. However, these hypotheses require further confirmatory experiments.

Usually, TGFβ acts as a tumor-suppressive factor because of its growth inhibitory effect on epithelial cells. Indeed, studies observed a pro-oncogenic function of TGFβ, especially in promoting migration, invasion, and metastasis of tumor cells [90,91,92]. Besides the well-known activation of the Smad pathway by binding of TGFβ to its corresponding receptor [93], TGFβ is able to mediate its effects via other pathways like the PI3K/AKT pathway [94]. Consistently, osteoblast-derived TGFβ1 activates AKT signaling in MDA-MB-231 cells [95]. Furthermore, activation of the AKT signaling pathway by TGFβ antagonizes the Smad pathway, for example, through direct binding of AKT to Smad3 or through AKT-dependent inhibition of FOXO [96,97,98]. Since TGFβ-stimulation was shown to increase CTGF expression in breast cancer cells, we propose that this is regulated at least in part via the PI3K/AKT3 pathway in an isoform-specific manner, because the diminished increase in CTGF expression could only be observed after AKT3 knockdown [19]. We suggest, on the one hand, that AKT3 is either a direct downstream effector of TGFβ-induced CTGF expression or cooperates and regulates the TGFβ/Smad/CTGF axis. Mechanistically, the promotor region of *Ctgf* bears a binding site for the nuclear factor kappa-light-chain-enhancer of activated B cells (NFκB) and AKT activates NFκB via degradation of the inhibitor of nuclear factor kappa-B (IκB) [99]. On the other hand, there are three possible mechanisms for AKT3 as a possible modulator of the TGFβ-activated Smad signaling. First, AKT mediated TWIST phosphorylation and activation, increasing expression of its transcriptional target TGFβ2 which, in turn, stimulated TGFβ signaling. Second, AKT directly stabilizes TβR1 through regulation of the deubiquitinating ubiquitin-specific protease 4. Third, AKT is able to inhibit GSK3β-mediated Smad3 instability [98].

The matricellular protein CTGF is upregulated in bone-metastasizing breast cancer cells, providing further evidence for the importance of CTGF in the vicious cycle of tumor-induced osteolysis [61]. Inhibition of CTGF with a neutralizing antibody in an MDA-MB-231 metastasis mouse model diminishes the formation of osteolytic bone metastases and the number of associated osteoclasts [100]. On the mechanistic level, a stimulatory effect of CTGF on osteoclastogenesis and bone resorption could be, in part, through an induction of dendritic cell-specific transmembrane protein (DC-STAMP) as an osteoclast differentiation factor. CTGF is able to directly bind to RANK and induces NFκB-mediated osteoclast differentiation. CTGF mediates its effect on osteolysis besides direct effects on osteoclasts via an upregulation of RUNX2 and secreted RANKL. Consistently, a strong positive correlation of CTGF and RANKL expression was reported in bone metastases of human breast cancer [61,101]. Beneath the paracrine effect of CTGF on osteoclasts and osteoblasts, we propose that CTGF could also bind to RANK on the breast cancer cells, stimulating the tumor cells in an autocrine manner. In addition to former studies reporting RANK expression in breast cancer cell lines as well as human breast cancer probes, we demonstrated here that RANK is expressed independently of AKT isoform knockdowns and TGFβ-stimulation in 231-BO bone-metastasizing breast cancer cells [62,102]. Kang et al. showed that overexpression of CTGF alone in breast tumor cells is not sufficient to increase osteolytic lesions in a mouse model. Only the combined overexpression of IL11, osteopontin, and CTGF was able to promote the formation of osteolytic lesions [19]. Thus, a decrease in TGFβ-stimulated CTGF expression might have only a minor effect on osteolysis and thus, results in only the mild effect of an absent elevation and not a significant decrease in osteolysis in the AKT3 knockdown of 231-BO cells.

Once bone metastases occur, treatment with bisphosphonates or denosumab remain the only option in this incurable stage. Hormone receptor and HER2 positive breast cancer shows a higher incidence of bone metastases, but triple negative breast cancer patients with bone metastases exhibit a lower median survival [6,7]. Furthermore, triple negative breast cancer lacks an approach for targeted therapy. This is why we used a bone-seeking triple negative breast cancer subline to study the function of AKT isoforms for bone metastasis. Targeting dysregulated intracellular signaling pathways such as the AKT signaling pathway constitutes a promising future therapeutical strategy in bone metastasis of breast cancer, since AKT is frequently dysregulated in triple negative breast cancer and bone metastasis of breast cancer [8,27,29]. As a consequence, the mTOR inhibitor Everolimus is already approved and used for treatment of advanced breast cancer [103]. The panAKT inhibitor Ipatasertib was already tested in a clinical trial in combination with paclitaxel and showed favorable results [104]. Additionally, MK2206 and AZD5363 were already tested in combination with other drugs such as doxorubicin in several clinical trials with promising results [105]. Using AKT inhibitors was shown to overcome resistance to anti-HER2 treatment in breast cancer cells [106]. On the basis of our data, we suggest considering an isoform-specific drug development as well as a stage-dependent drug application. Inhibition of AKT3 in an early stage of breast cancer without metastases or at the beginning of the metastatic cascade seems not expedient, whereas in patients with already developed bone metastases, inhibition of AKT3 could be advantageous. Consequently, treatment of mice with MK2206 after inoculation of breast cancer cells results in an enhanced metastasis to lung [107]. On the basis of our data, the treatment of triple negative breast cancer in a non-metastatic stage with an AKT1/2 inhibitor [108] in combination with other established drugs or mTOR inhibitors like RAD001 should be taken forward in further studies.

## 5. Conclusions

Bone metastasis is a frequent phenomenon in breast cancer and is related with a lack of appropriate antitumor therapy. Since the AKT signaling plays a crucial role in the bone metastasis of breast cancer, we investigated the functional role of the individual AKT isoforms in a bone-metastasizing breast cancer cell line. We revealed an enhanced AKT3 activity in these cells but shRNA-mediated knockdown of AKT3 resulted in an increased migration, invasion, and chemotaxis in vitro as well as metastasis to bone in vivo. Nevertheless, despite a higher tumor burden in bone, inoculation of 231-BO cells lacking AKT3 did not significantly promote osteolysis. Consistently, we reported an increased activity and phosphorylation of pro-metastatic HER2 and DDR1/2 in the 231-BO AKT3 knockdown cells, whereas TGFβ-stimulated and osteolysis-associated CTGF expression was impaired. Based on the reported response of 231-BO cells to combined AKT/mTOR inhibition in vitro, we suggest that an isoform-specific inhibition of AKT could be a promising therapeutical approach in bone metastasis of breast cancer.

## Data Availability

The datasets used and/or analyzed during the current study are available from the corresponding author on reasonable request.

## Supplementary Materials

The following are available online at https://www.mdpi.com/2073-4409/10/2/430/s1, Figure S1: Combined treatment of 231-BO cells with MK2206 and RAD001 exhibits synergistic effects on proliferation; Figure S2: Phosphorylation of AKT isoforms in 231-BO cells; Figure S3: Images of migration wound healing assays of 231-BO cells with AKT isoform knockdown; Figure S4: Images of invasion wound healing assays of 231-BO cells with AKT isoform knockdown; Figure S5: MMP2 is upregulated in 231-BO AKT2 knockdown cells; Figure S6: Images of bioluminescence measurement of NSG mice injected with 231-BO cells harboring AKT isoform knockdowns; Figure S7: Knockdown of AKT1 in 231-BO cells decreases metastasis to adrenal glands after intracardiac inoculation into NSG mice; Figure S8: Knockdown of AKT3 in 231-BO cells has no effect on osteolysis after intracardiac injection into NSG mice; Figure S9: S100A4 is exclusively expressed in the MDA-MB-231 parental cell line.
