# Supplementary material for: Knockdown of AKT3 Activates HER2 and DDR Kinases in Bone-Seeking Breast Cancer Cells, Promotes Metastasis In Vivo and Attenuates the TGFβ/CTGF Axis
Source: Cells. 2021 Feb 18;10(2):430. doi: 10.3390/cells10020430 (PMC7922044; doi:10.3390/cells10020430)
Supplement: Supplementary file 1 [file cells-10-00430-s001.pdf]

A

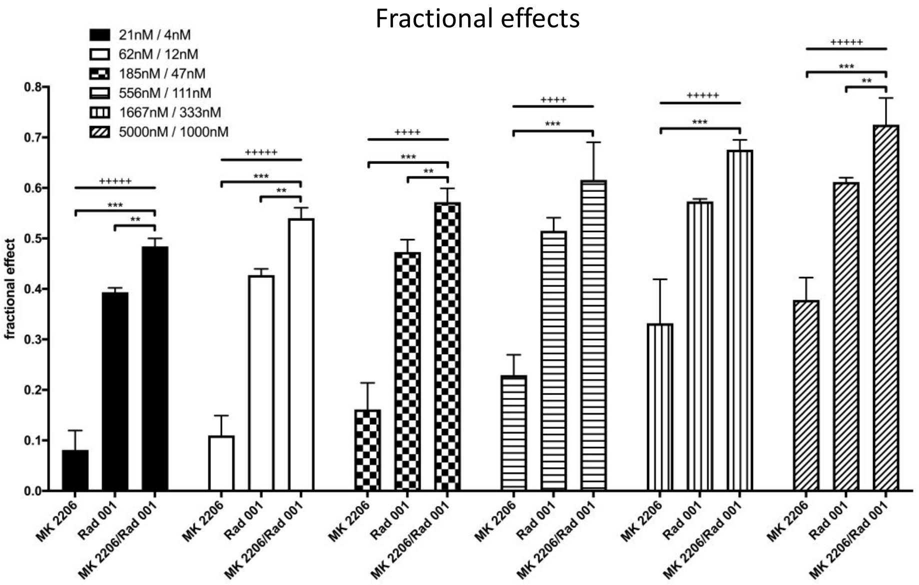

B

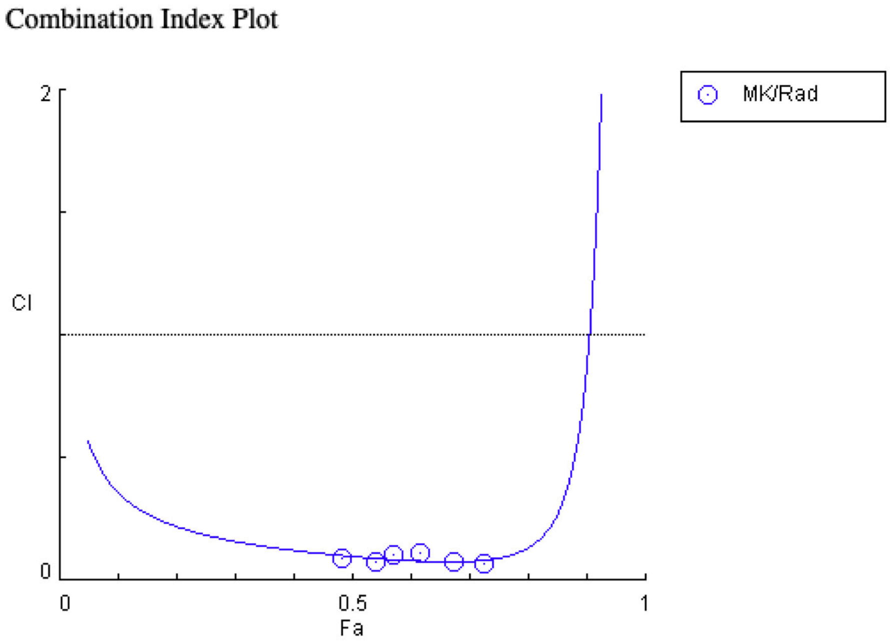

Figure S1. Combined treatment of 231-BO cells with MK2206 and RAD001 exhibits synergistic effects on proliferation. (**A**) Fractional effects of treatment with RAD001 and MK 2206 either alone or in combination were calculated from Alamar Blue Assay relative to DMSO control. Combination indices (CI) were determined according to the Chou and Talalay method (++++ = CI 0.1 – 0.3, strong synergism; +++++ = CI < 0.1, very strong synergism). Bars indicate mean with SD; ** = $p < 0.01$; *** = $p < 0.001$. (**B**) The graph represents the combination index plot for combined treatment with MK2206 and RAD001 of 231-BO cells. Combined treatment of 231-BO cells with MK2206 and RAD001 showed synergistic effects in all tested concentrations.

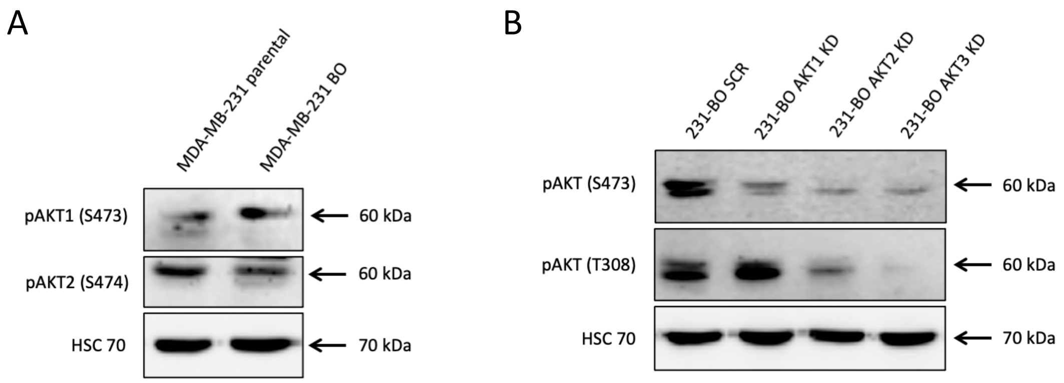

Figure S2. Phosphorylation of AKT isoforms in 231-BO cells. (**A**) Level of pAKT1 S473 and pAKT2 S474 in 231-BO cells and parental MDA-MB-231 cells were determined by Western blot analysis using isoform-specific phospho-antibodies. HSC70 functions as a loading control. (**B**) Level of pAKT T308 and pAKT S473 in 231-BO cells with AKT isoform knock-downs were analyzed by Western blot analysis compared to SCR control. HSC70 functions as a loading control.

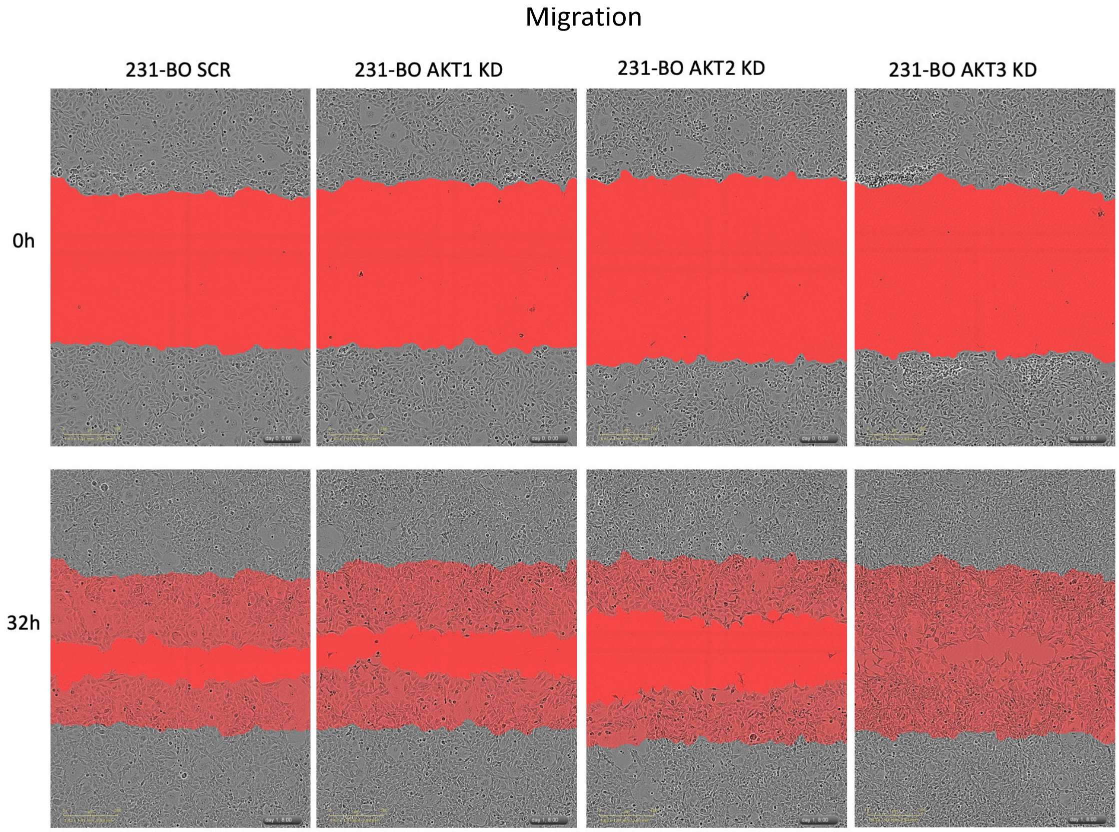

Figure S3. Images of migration wound healing assays of 231-BO cells with AKT isoform knockdown. Migration of 231-BO cells lacking individual AKT isoforms was tested using wound healing scratch assay of nearly confluent monolayers. Images were taken with the IncuCyte live cell imaging systems and representative images were chosen. Different red color intensities represent initial scratch mask and actual scratch mask analyzed with the IncuCyte Zoom software.

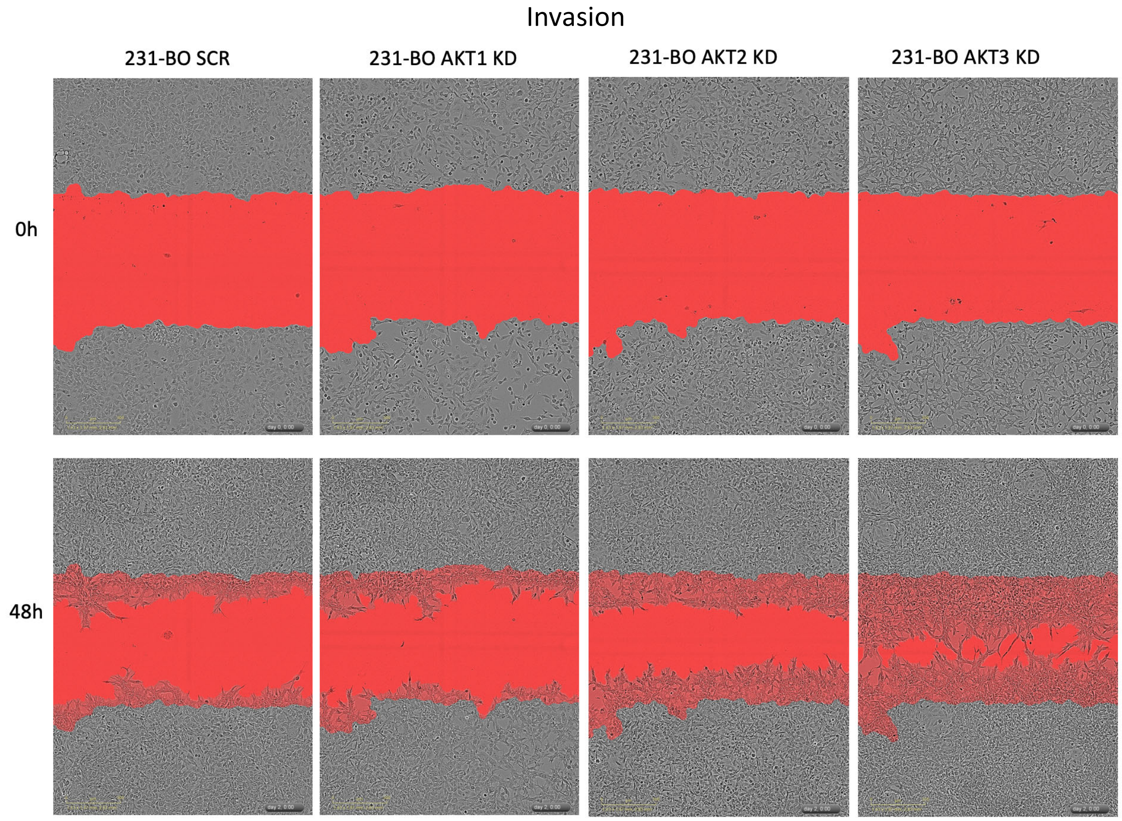

Figure S4. Images of invasion wound healing assays of 231-BO cells with AKT isoform knockdown. Invasion of 231-BO cells with AKT isoform knockdown was tested using wound healing scratch assay of nearly confluent monolayers. Matrigel was added as an ECM after scratches were generated. Images were taken with the IncuCyte live cell imaging systems and representative images were chosen. Different red color intensities represent initial scratch mask and actual scratch mask analyzed with the IncuCyte Zoom software.

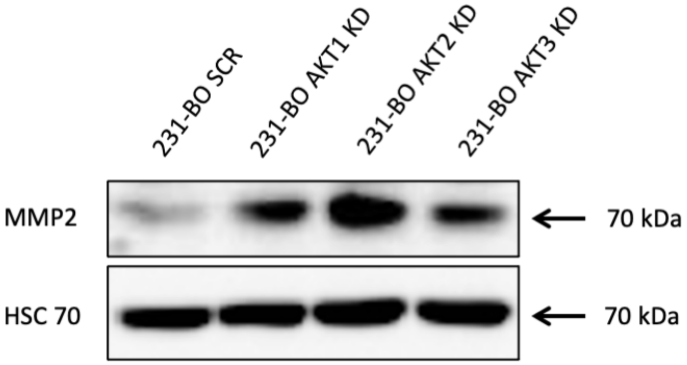

Figure S5. MMP2 is upregulated in 231-BO AKT2 knockdown cells. MMP2 expression in 231-BO cells with AKT isoform knockdown was analyzed by Western blot analysis compared to SCR control. Antibodies directed against the indicated proteins were used. HSC70 functions as a loading control.

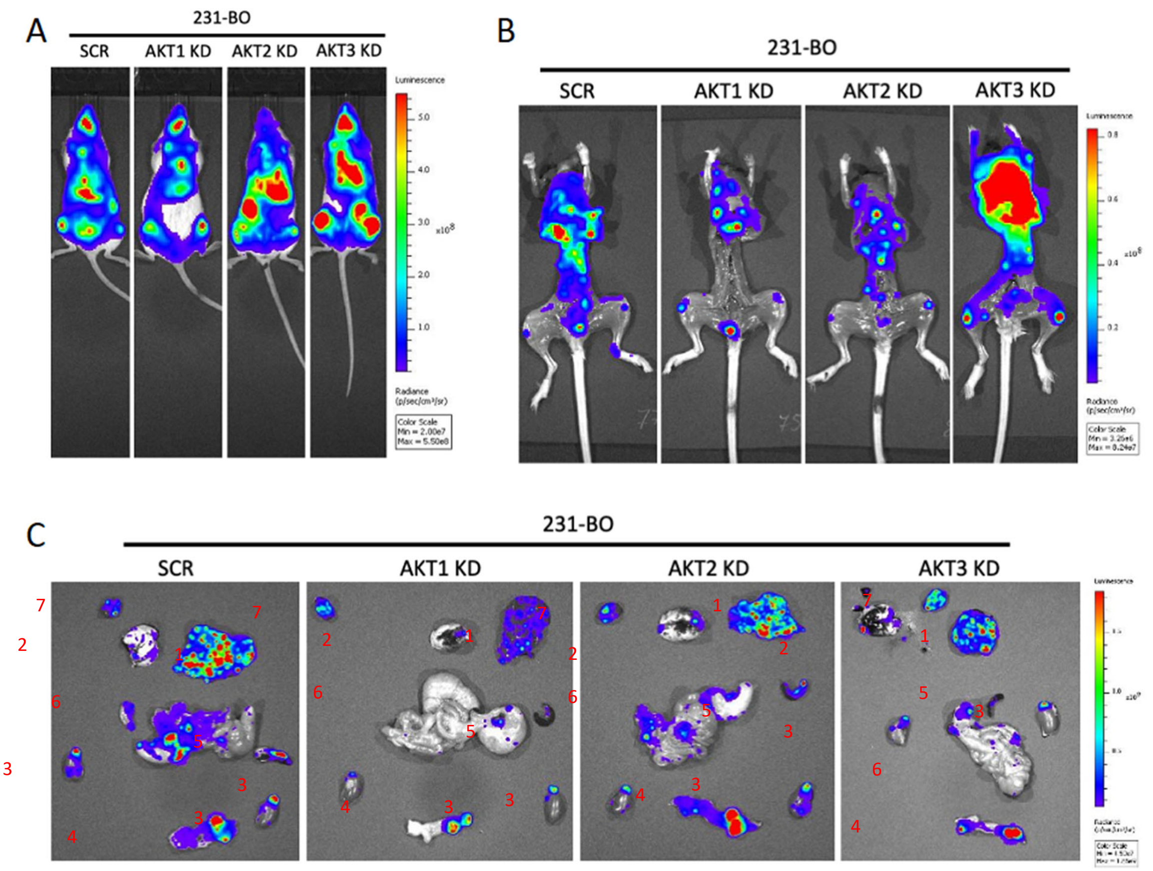

Figure S6. Images of bioluminescence measurement of NSG mice injected with 231-BO cells harboring AKT isoform knockdowns. AKT isoform knockdown 231-BO cells transduced with luciferase were inoculated into the left ventricle of NSG mice. Mice were sacrificed after 21 days, were exenterated and organs as well as skeletal systems were collected. Representative images of bioluminescence measurements of whole mice in vivo (**A**), skeletal system ex vivo (**B**) and organs ex vivo **(C)** with an IVIS imaging system are shown (1: liver, 2: lungs, 3: kidneys with adrenal glands, 4: ovaries, 5: spleen, 6: intestine, 7: heart).

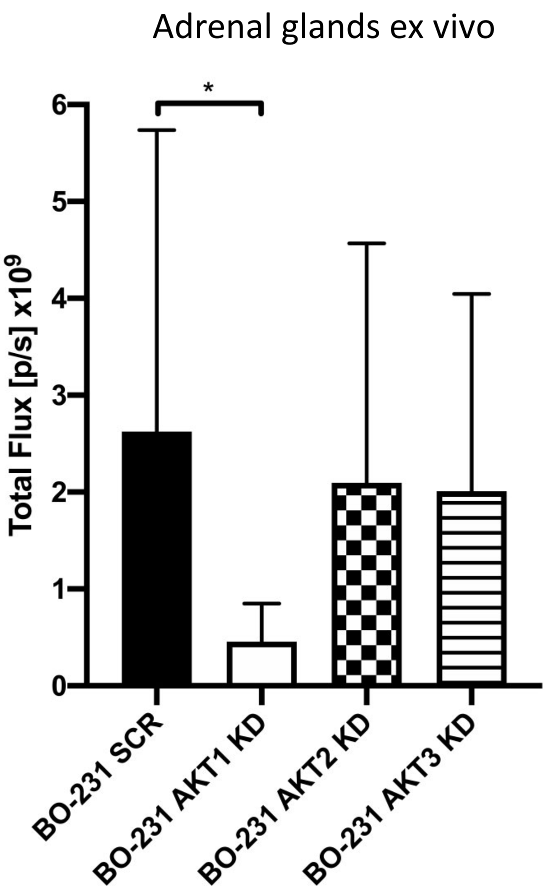

Figure S7: Knockdown of AKT1 in 231-BO cells decreases metastasis to adrenal glands after intracardiac inoculation into NSG mice. 231-BO cells with AKT isoform knockdowns were transduced with a luciferase vector and were inoculated into the left ventricle of NSG mice. Mice were sacrificed after 21 days, exenterated and organs were harvested. Bioluminescence intensities of adrenal glands ex vivo were measured by an IVIS imaging system (SCR and AKT1 KD: n = 16; AKT2 KD and AKT3 KD n = 18). Bars indicate mean with SD; * = $p < 0.05$.

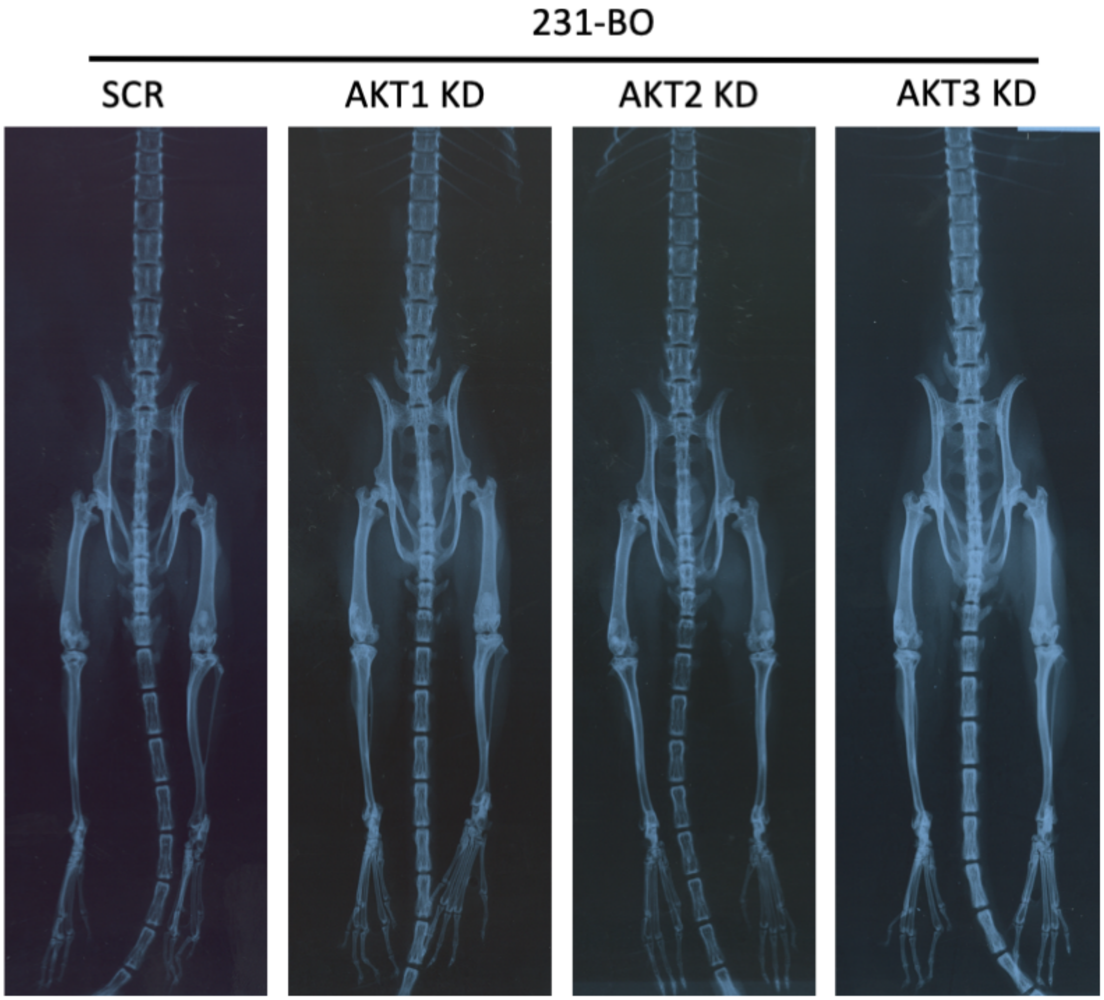

Figure S8. Knockdown of AKT3 in 231 BO cells has no effect on osteolysis after intracardiac injection into NSG mice. Skeletal systems of NSG mice injected with 231-BO cells with AKT isoform knockdowns were harvested after mice were sacrificed. Radiographic images of skeletal systems ex vivo were taken and presence of osteolysis in spine, hind limbs or pelvis was analyzed.

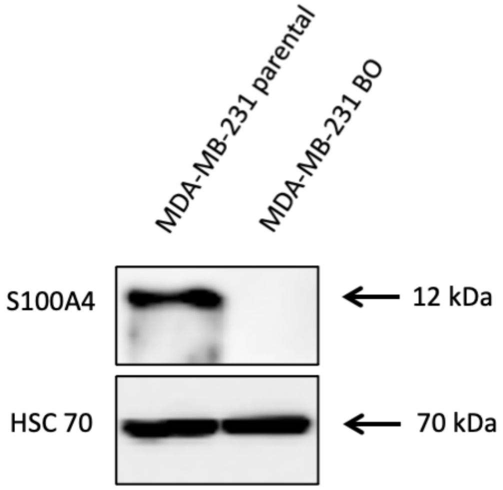

Figure S9. S100A4 is exclusively expressed in the MDA-MB-231 parental cell line. Expression of S100A4 as a pro-migratory protein was analyzed by Western blot analysis of bone-seeking 231-BO cells compared to MDA-MB-231 parental cells. Antibodies directed against the indicated proteins were used. HSC70 functions as a loading control.
